# Senescent Cells Involved in Deterioration of Bone Microstructure by High‐Frequency Parathyroid Hormone 1–34 Administration and Bone Loss

**DOI:** 10.1111/acel.70331

**Published:** 2025-12-23

**Authors:** Masayuki Bun, Yuichiro Ukon, Masato Ikuta, Takayuki Kitahara, Takuya Furuichi, Hiromasa Hirai, Daisuke Tateiwa, Yuya Kanie, Masayuki Furuya, Takahito Fujimori, Satoru Otsuru, Seiji Okada, Takashi Kaito

**Affiliations:** ^1^ Department of Orthopaedic Surgery Osaka University Graduate School of Medicine Osaka Japan; ^2^ Department of Orthopaedic Surgery Osaka International Medical and Science Center Osaka Japan; ^3^ Department of Orthopaedic Surgery University of Maryland College Park Maryland USA; ^4^ Department of Orthopaedic Surgery Osaka Rosai Hospital Osaka Japan

**Keywords:** bone loss, cellular senescence, osteoblast, osteoporosis, parathyroid hormone

## Abstract

Osteoporosis is characterized by reduced bone mass and structural deterioration, leading to increased fracture risk, particularly in older adults. Parathyroid hormone (PTH) is a widely used anabolic therapy for osteoporosis; however, rapid bone loss after treatment discontinuation presents a significant clinical challenge. Cellular senescence has been implicated in age‐related bone fragility. However, its role in PTH‐induced bone remodeling and post‐treatment bone loss remains unclear. This study aimed to investigate the effects of PTH administration frequency on bone microarchitecture and cellular senescence in young and aged mice. High‐frequency PTH administration improved trabecular bone volume in both age groups, but caused cortical bone thinning, increased porosity, and elevated osteoclast activity in aged mice. PTH induces senescent osteoblast‐lineage‐enriched cell accumulation in aged, but not young mice, accompanied by upregulation of senescence‐associated markers and activation of the mechanistic Target of Rapamycin Complex 1 pathway. Co‐administration of the senolytic agents dasatinib and quercetin during PTH treatment reduced senescent cell burden, improved cortical porosity, and mitigated rapid bone loss after PTH discontinuation in aged mice. These findings indicate that senescent osteoblast‐lineage‐enriched cells contribute to bone fragility and post‐treatment bone loss in aged individuals, suggesting that targeting senescence may enhance the efficacy and sustainability of PTH therapy for osteoporosis.

## Graphical abstract

Senescent osteoblasts induced by PTH contribute to cortical bone loss in aged mice. Senolytic co‐treatment reduces senescence and prevents bone loss after PTH withdrawal, enhancing the durability of osteoporosis therapy.

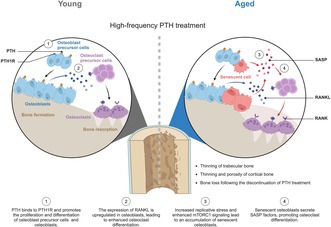

## 1 Introduction

Osteoporosis, which is characterized by a reduction in bone mineral density and deterioration of bone structure, represents a significant public health challenge, particularly in older adults (Johnell and Kanis 2006). Intermittent administration of parathyroid hormone (PTH) 1–34 has emerged as a pivotal therapeutic approach for osteoporosis, primarily because of its strong anabolic effects on bone metabolism (Nakamura et al. 2012; Neer et al. 2001). PTH increases bone mineral density by promoting bone remodeling, in which bone formation is favored over resorption (Chiba et al. 2022). However, the challenge in treating osteoporosis is the rapid bone loss that occurs after discontinuation of the bone anabolic treatment, and PTH is no exception (Crandall et al. 2014; Leder et al. 2009). The mechanism underlying rapid bone loss following PTH discontinuation remains incompletely elucidated (Singh et al. 2022; Tseng et al. 2022; Wang et al. 2022).

Recent studies of bone metabolism have highlighted the role of cellular senescence in the development of age‐related osteoporosis (Farr et al. 2017; Zhang et al. 2022). With aging, senescent cells (osteoblasts and osteocytes), characterized by irreversible cell cycle arrest and changes in the secretory phenotype (senescence‐associated secretory phenotype [SASP]), accumulate in the bone microenvironment. Inflammatory cytokines secreted by senescent cells promote osteoclast formation and inhibit osteoblast mineralization, causing bone quality deterioration and bone fragility (Farr et al. 2017). Targeting these cells can exert antiresorptive and anabolic effects on bones (Farr and Khosla 2019). Despite these insights, it is unclear whether PTH administration affects cellular senescence in bone or whether the accumulation of senescent cells induced by PTH is related to bone resorption after the discontinuation of PTH administration.

This study aimed to evaluate the effect of PTH treatment on cellular senescence in the bone microenvironment of young and aged mice and to elucidate the relationship between changes in senescent cells in the bone microenvironment and bone loss after discontinuation of PTH administration. The findings of this study may provide new insights into optimizing PTH treatment for patients with osteoporosis by targeting cellular senescence in the bone microenvironment.

## 2 Results

### 2.1 Bone Morphology Showed Changes by PTH Administration

**FIGURE 1:**
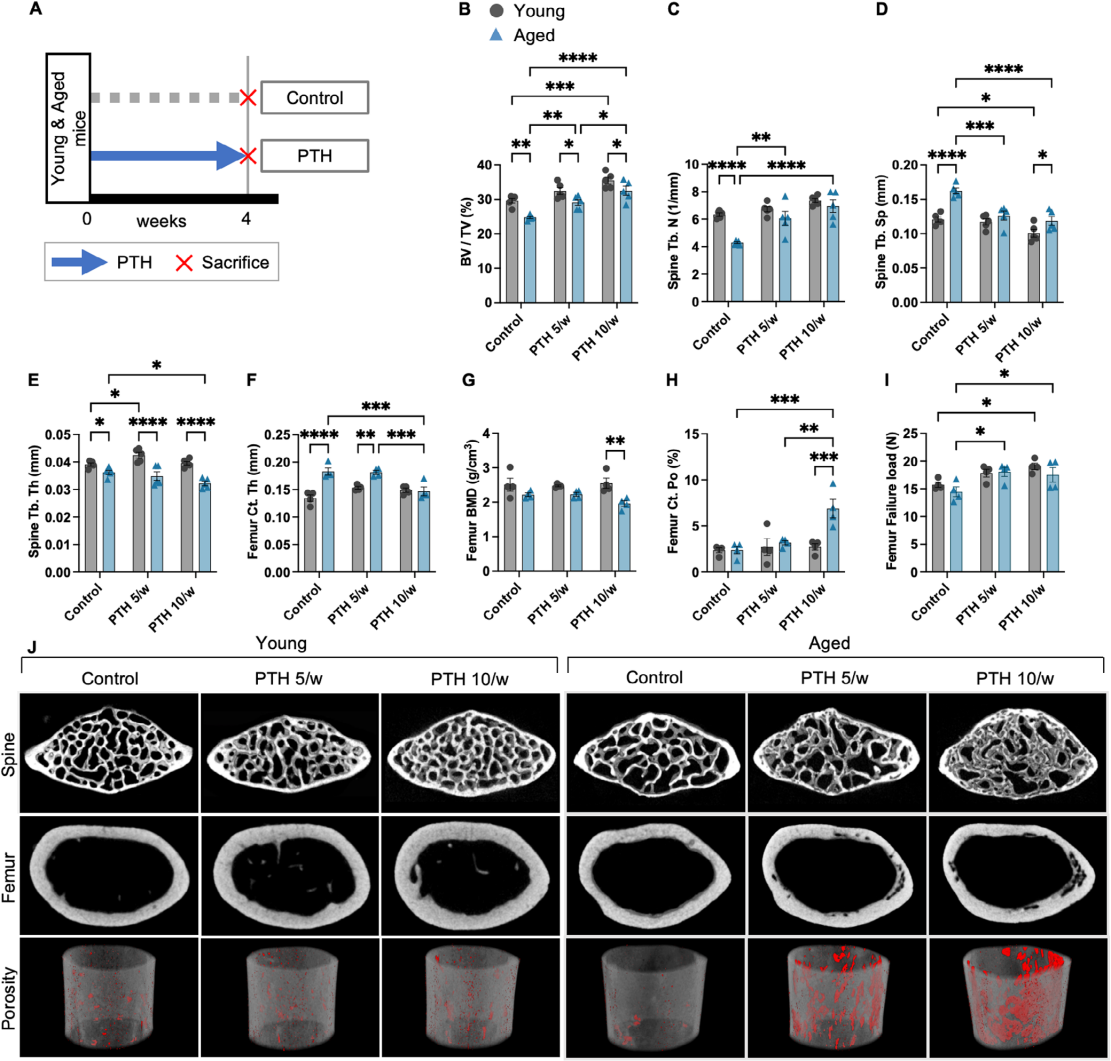
Effects of parathyroid hormone treatment frequency on bone morphology in young and aged mice. (A) Schematic illustration of the experimental design. Representative micro‐CT analysis of the lumbar spine and femur of C57BL/6 mice treated with PTH and vehicle (*n* = 5 mice/treatment). Quantitative analysis of trabecular bone volume (BV/TV) (B), trabecular thickness (Tb. Th) (C), trabecular number (Tb. N) (D), and trabecular spacing (Tb. Sp) (E), in the lumbar spine. (F) Micro‐CT images of the lumbar spine and femur. Three‐dimensional reconstructed image of cortical bone with porosity highlighted in red (below). Analysis of cortical thickness (Ct. Th) (G), cortical bone mineral density (Ct. BMD) (H), and cortical porosity (Ct. Po) (I). (J) Mechanical testing result for the failure load of the femur. Data represent mean ± SEM (error bars). *p < 0.05; **p < 0.01; ***p < 0.001; ****p < 0.0001 (two‐way ANOVA with Tukey's multiple comparisons test). PTH, parathyroid hormone; SEM, standard error of the mean; CT, computed tomography.

Both aged and young mice received PTH administration at two distinct frequencies (low‐frequency [5 times/week] and high‐frequency [10 times/week], 200 μg/kg/week for 4 weeks) to clarify the differences in the effects of PTH on bone microstructure depending on the aging status of the mice (Figure 1A, Table 1). Bone turnover in rats is two to three times higher than that in humans (Takakura et al. 2016; Yamamoto et al. 2016). Thus, PTH was administered to mice 10 times a week to mimic the daily PTH administration and five times a week to simulate the weekly PTH regimen in clinical settings in humans.

**TABLE 1:** Dosing frequency and daily dosage of parathyroid hormone administration.

Groups	Treatment	Dosage in 1 injection (μg/kg per Dose)	Dosing	Total weekly dose (μg/kg per week)
Control	Vehicle	0	5 times a week	0
PTH 5/w	PTH	40	5 times a week	200
PTH 10/w	PTH	20	10 times a week	200

Abbreviation: PTH, parathyroid hormone.

In both aged and young mice, an increased frequency of PTH administration led to a similar increase in bone volume fraction (BV/TV) and trabecular number (Tb.N) while decreasing trabecular separation (Tb.Sp) (Figure 1B–D). However, in aged mice, high‐frequency PTH administration was associated with a decrease in trabecular and cortical bone thickness (Ct.Th) and cortical bone mineral density (BMD), and a significant increase in cortical bone porosity (Ct.Po) (Figure 1E–H, Figure S1).

In contrast, in young mice, low‐frequency PTH administration resulted in trabecular bone thickening, whereas high‐frequency administration did not lead to thinning of either trabecular or cortical bone. In addition, even with high‐frequency PTH administration, no cortical bone porosity was observed in the femoral diaphysis, and bone mineral density (BMD) remained unchanged (Figure 1E–H, Figure S1). Mechanical testing was performed to assess whether these quantitative changes correlated with alterations in bone strength. It revealed that low‐frequency PTH administration increased the failure load in both aged and young mice; in contrast, high‐frequency PTH administration further improved the failure load only in young mice but not in aged mice (Figure 1I). These results show that the effect of PTH on bone microstructure and strength differed between young and aged mice.

### 2.2 PTH Administration Significantly Increased Bone Resorption Markers

**FIGURE 2:**
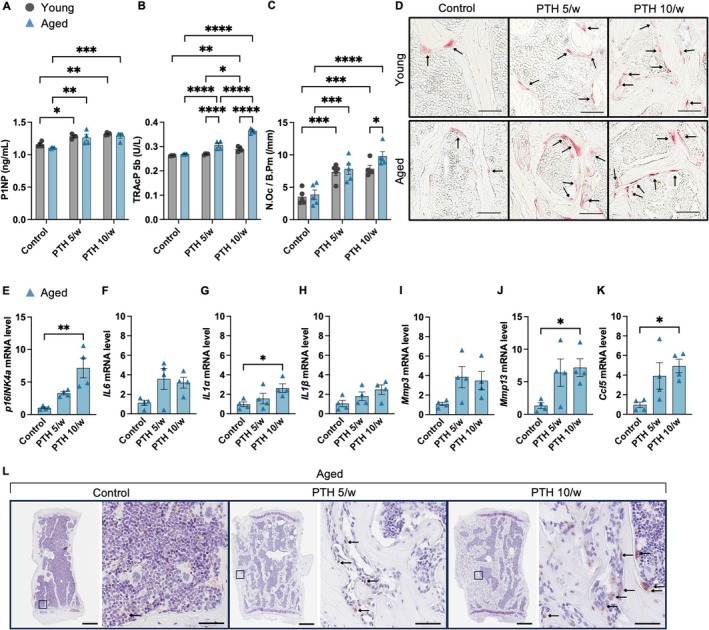
Changes in bone metabolism markers, distribution of osteoclasts, and accumulation of senescent cells in response to parathyroid hormone administration. Serum bone metabolism markers (*n* = 4) were analyzed. (A) The bone formation marker, amino‐terminal propeptide of type I collagen (P1NP, ng/mL). (B) Bone resorption marker tartrate‐resistant acid phosphatase 5b (TRAcP5b; U/L). (C) Number of TRAP‐positive osteoclasts per bone perimeter (N. Oc/B. Pm; 1/mm) (*n* = 5). (D) Distribution of TRAP‐positive osteoclasts in the spines of young and aged mice treated with different PTH administration frequencies. RT‐qPCR analysis of mRNA expression levels of *p16INK4a* (E) and SASP factors (*IL6*, *IL1α*, *IL1β*, *Mmp3*, *MMP13*, *Ccl5*) (F–K) in osteoblast and osteocyte‐enriched cells derived from the bones of aged mice (*n* = 4 mice/treatment). (L) IHC for p16INK4a in the lumbar spine of aged mice (see arrows, scale bars, 500 μm; 50 μm on right). Data represent mean ± SEM (error bars). *p < 0.05; **p < 0.01; ***p < 0.001; ****p < 0.0001 (one‐ or two‐way ANOVA with Tukey's multiple comparisons test). PTH, parathyroid hormone; TRAP, tartrate‐resistant acid phosphatase; SEM, standard error of the mean; ANOVA, analysis of variance; IHC, immunohistochemistry; SASP, senescence‐associated secretory phenotype; *p16INK4a*, cyclin‐dependent kinase inhibitor 2A; *IL6*, Interleukin‐6; *IL1α*, Interleukin‐1 alpha; *IL1β*, Interleukin‐1 beta; *Mmp3*, Matrix metalloproteinase‐3; *MMP13*, Matrix metalloproteinase‐13; *Ccl5*, *C‐C motif chemokine ligand 5*.

Given the observed differences in the bone microstructure between young and aged mice after PTH administration, serum bone metabolism markers were investigated. Amino‐terminal propeptide of type I collagen (P1NP) and tartrate‐resistant acid phosphatase 5b (TRAcP5b) levels were elevated after the 4‐week PTH treatment in both aged and young mice; however, in aged mice, the increase in TRAcP5b was significantly greater than that in young mice (Figure 2A,B). Tartrate‐resistant acid phosphatase (TRAP) staining showed that the increase in the number of osteoclasts due to the increased frequency of PTH administration was greater in aged mice than in young mice (Figure 2C,D). These findings were consistent with the results of osteoblast surface per bone surface (Ob.S/BS) and osteoclast surface per bone surface (OC.S/BS) as assessed by hematoxylin and eosin (HE) staining (Figure S2A–C). These results show that the activity of osteoclasts is enhanced by PTH administration in aged mice compared to that in young mice.

### 2.3 Senescent Cell Accumulation in Bone by PTH Administration

Next, we investigated the association between bone microstructural changes induced by PTH administration and the accumulation of senescent cells in aged and young mice, using polymerase chain reaction (PCR) and immunohistochemistry (IHC). PTH administration induced cellular proliferation, as evidenced by Ki‐67‐positive staining, predominantly in mesenchymal stem cells (MSC) and osteoblast‐lineage‐enriched cells in both young and aged mice (Figure S3C). In aged mice, the expression levels of *p16INK4a* (cyclin‐dependent kinase inhibitor 2A) and SASP factor genes—*IL1α* (interleukin‐1 alpha), *MMP13* (matrix metallopeptidase 13), and *Ccl5* (C‐C motif chemokine ligand 5)—were significantly increased by PTH administration in a frequency‐dependent manner (Figure 2E–K). IHC staining for p16INK4a showed increased localization of senescent cells in the high‐frequency PTH group, with prominent accumulation in regions adjacent to trabeculae (Figure 2L).

In contrast, in young mice, the number of senescent cells and the expression levels of *p16INK4a* and *SASP* genes were not increased by PTH administration (Figure S4A–I). These results revealed distinct differences in senescent cell accumulation between aged and young mice in response to PTH administration.

### 2.4 PTH Activated the Mechanistic Target of Rapamycin Complex 1 Pathway in Osteoblasts In Vitro

**FIGURE 3:**
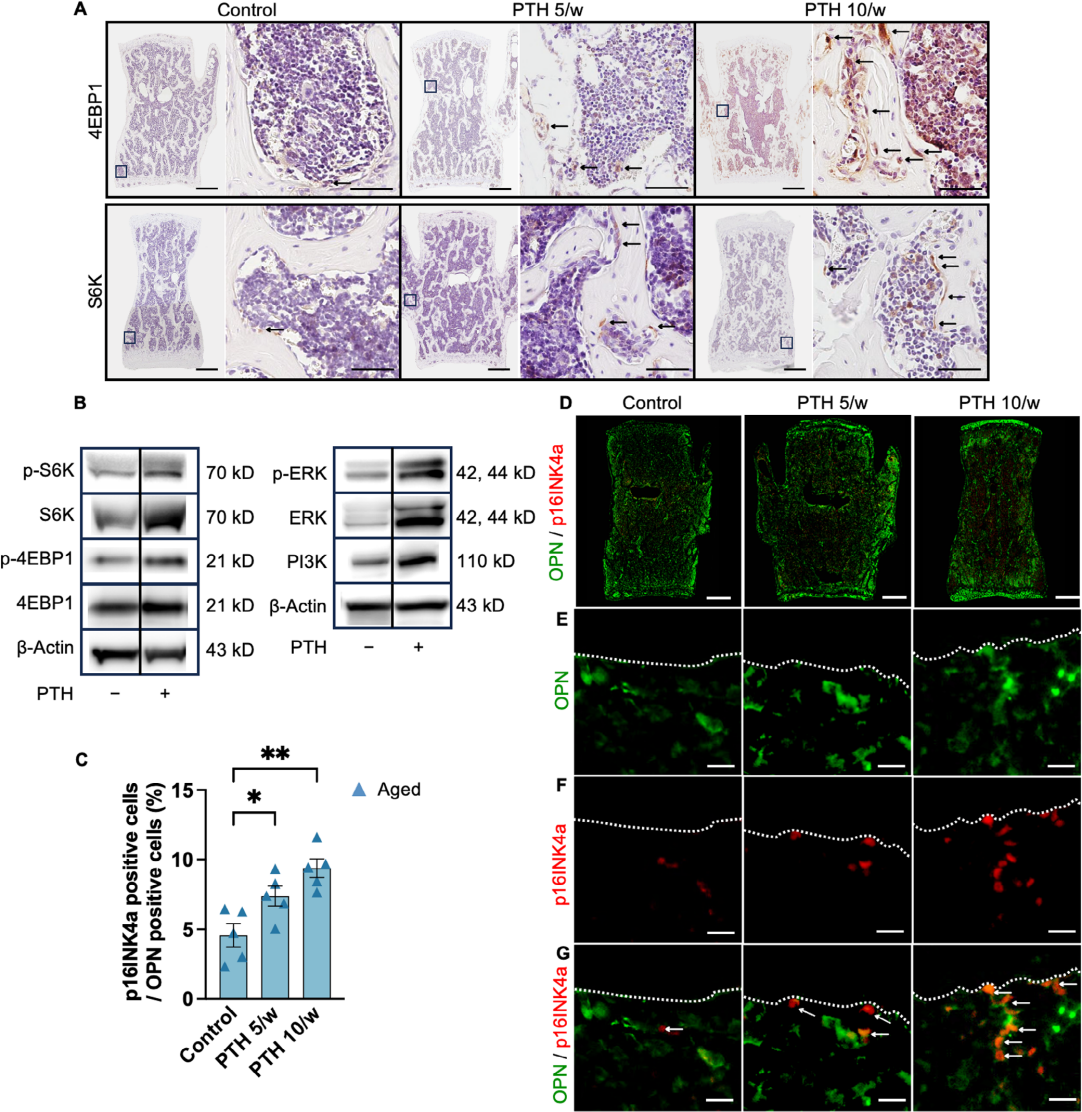
Parathyroid hormone treatment induced senescent osteoblasts through the mammalian target of rapamycin complex 1 pathway. (A) IHC for 4EBP1 and S6K in the lumbar spine (scale bars, 500 μm; 50 μm on the right). The arrow indicates the positive staining of 4EBP1 or S6K. (B) Western blot analysis of 4EBP1, S6K, ERK, and PI3K. Osteoblasts were treated with 100 nM PTH for 24 h and harvested for protein isolation. (C) Percentage of p16INK4a‐positive cells among OPN‐positive cells (*n* = 5). (D–G) Double immunofluorescence staining for p16INK4a (red) and OPN (green) in the lumbar spine of aged mice. The arrow indicated the positive staining of p16INK4a and OPN. Scale bars, D: 10 μm, (E–G) 10 μm. Data represent mean ± SEM (error bars). *p < 0.05; **p < 0.01 (one‐way ANOVA with Tukey's multiple comparisons test). PTH, parathyroid hormone; SEM, standard error of the mean; ANOVA, analysis of variance; IHC, immunohistochemistry; 4EBP1, eukaryotic translation initiation factor 4E‐binding protein 1; S6K, S6 kinase; ERK, extracellular signal‐regulated kinase; PI3K, phosphoinositide 3‐kinase; OPN, osteopontin; *p16INK4a*, cyclin‐dependent kinase inhibitor 2A.

To directly investigate the relationship between PTH administration and senescent cell accumulation, mouse calvarial osteoblasts were treated with PTH. Given that the mechanistic Target of Rapamycin Complex 1 (mTORC1) is a pivotal promoter of cellular senescence, IHC was performed to detect the downstream markers of the mTORC1 pathway, including phosphorylated S6 kinase (p‐S6K) and phosphorylated eukaryotic translation initiation factor 4E‐binding protein 1 (p‐4EBP1), to confirm their activation following high‐frequency PTH administration (Figure 3A). Western blot analyses further demonstrated the direct effect of PTH on mTORC1 activity in osteoblasts, as evidenced by the phosphorylation of S6K and 4EBP1 under PTH treatment with a concentration of 100 nM (Figure 3B). Consistent with previous reports, phosphorylation of extracellular signal‐regulated kinase (ERK) and phosphoinositide 3‐kinase (PI3K) upregulation were observed upstream of mTORC1 (Marino and Bellido 2024; Martin et al. 2021; Yamamoto et al. 2007). These findings suggest that an increased frequency of PTH administration in old mice, where aging stresses such as replicative stress accumulation can induce osteoblast senescence through the activation of the mTORC1 pathway (Herr et al. 2024).

### 2.5 Senescent Osteoblast‐Lineage‐Enriched Cells Were Increased by PTH Administration in Aged Mice

Senescent osteoblasts are involved in bone remodeling and cortical bone thinning (Ukon et al. 2024). The observed changes in bone microstructure and the localization of senescent cells adjacent to trabeculae in aged mice following PTH administration suggest that PTH administration promotes the accumulation of senescent osteoblasts.

To verify that the senescent cells that accumulated in the bone following PTH administration were osteoblast‐lineage‐enriched cells, double immunostaining was performed on osteopontin (OPN) and p16INK4a. The results confirmed a significant increase in the number of OPN‐ and p16INK4a‐positive cells after high‐frequency PTH administration. These findings suggest that PTH administration induced osteoblast‐lineage‐enriched senescence in aged mice and that the accumulation of senescent osteoblast‐lineage‐enriched cells may be related to different responses to PTH treatment between aged and young mice.

### 2.6 Senolytics Therapy Improved Cortical Porosity in PTH‐Treated Aged Mice

**FIGURE 4:**
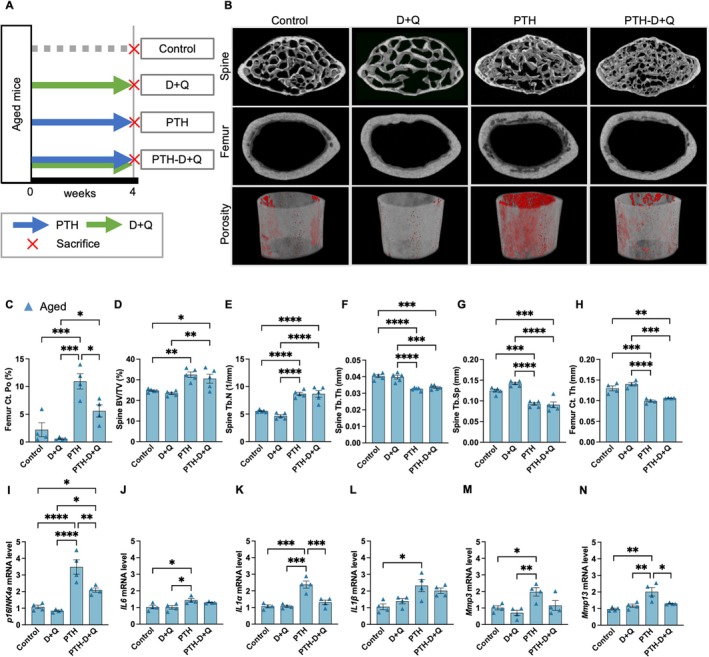
Effects of senolytic treatment (dasatinib + quercetin) on parathyroid hormone‐induced senescence and bone morphology. (A) Experimental design for PTH treatment (10 times a week) combined with D + Q in aged mice. (B) Representative micro‐CT images of the lumbar spine and femur. Cortical porosity is highlighted in red (below). Quantitative analysis of trabecular bone parameters (BV/TV, Tb. N, Tb. Sp, Tb. Th) in aged mice (*n* = 5 mice/treatment) (C–F), cortical thickness (Ct. Th) (G), and cortical porosis (Ct. Po) (H). (I–N) RT‐qPCR results indicating a reduction in *p16INK4a* and SASP factors (*IL6*, *IL1α*, *IL1β*, *Mmp3*, *MMP13*) (*n* = 4 mice/treatment). Data represent mean ± SEM (error bars). *p < 0.05; **p < 0.01; ***p < 0.001; ****p < 0.0001 (one‐way ANOVA with Tukey's multiple comparisons test). PTH, parathyroid hormone; D + Q, dasatinib + quercetin; CT, computed tomography; BV/TV, bone volume; Tb. N, trabecular number; Tb. Th, trabecular thickness; Tb. Sp, trabecular spacing; Ct. Th, cortical thickness; SASP, senescence‐associated secretory phenotype; SEM, standard error of the mean; ANOVA, analysis of variance; *p16INK4a*, cyclin‐dependent kinase inhibitor 2A; *IL6*, Interleukin‐6; *IL1α*, Interleukin‐1 alpha; *IL1β*, nterleukin‐1 beta; *Mmp3*, Matrix metalloproteinase‐3; *MMP13*, Matrix metalloproteinase‐13.

**FIGURE 5:**
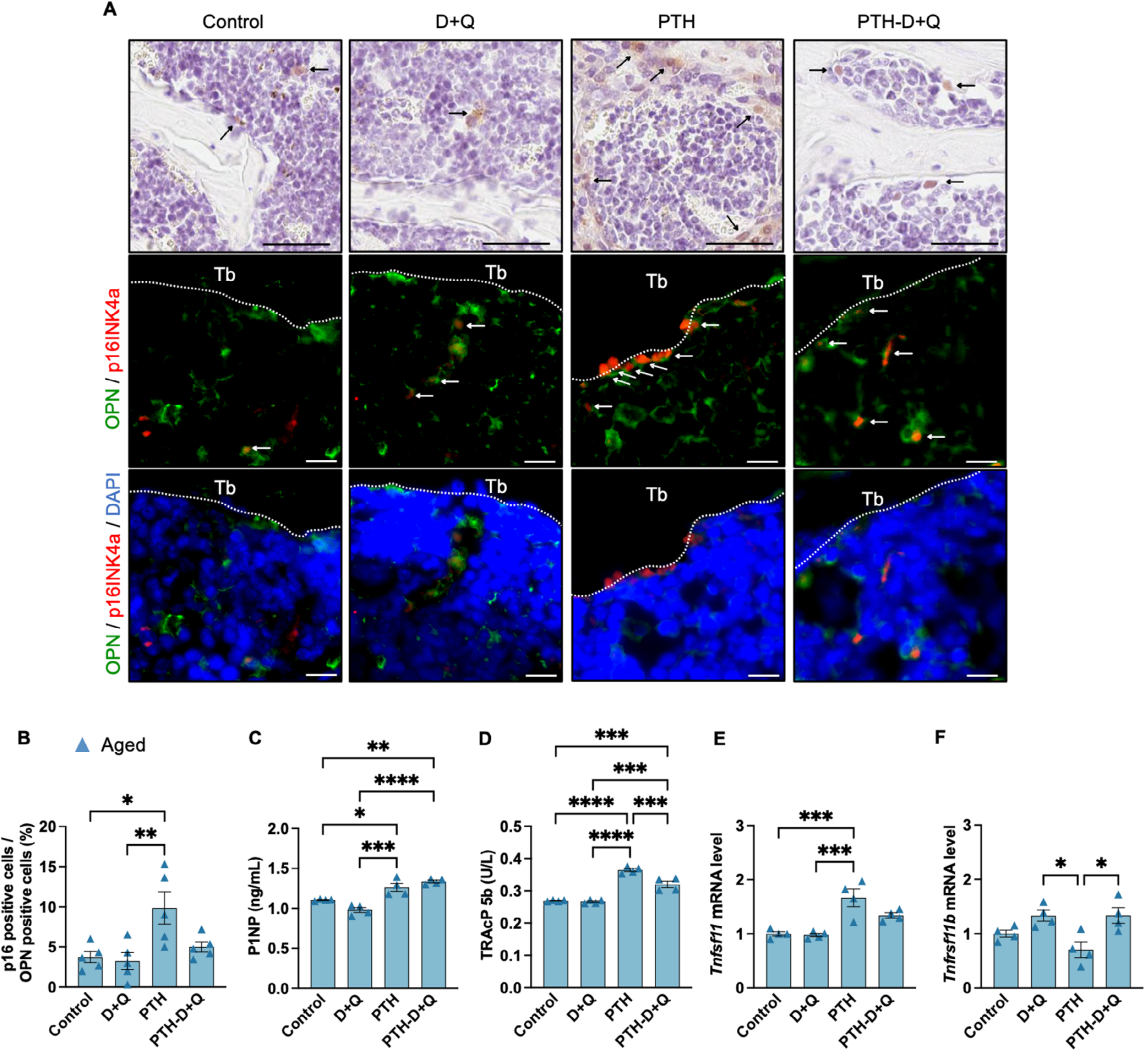
Effects of senolytic treatment (dasatinib + quercetin) on parathyroid hormone‐induced senescence and bone metabolic markers. PTH treatment (10 times per week) was combined with D + Q for 4 weeks in aged mice. (A) IHC for p16INK4a in the lumbar spine (see arrows). Scale bars, 50 μm (above). Double immunofluorescence staining of p16INK4a and OPN in the lumbar spine. The arrow indicates positive p16INK4a and OPN expression. Scale bars, 10 μm. (B) Percentage of p16INK4a‐positive cells among OPN‐positive cells (*n* = 5). (C) Bone formation marker P1NP (ng/mL) (*n* = 5). (D) The bone resorption marker, TRAcP5b (U/L) (*n* = 5). (E, F) RT‐qPCR results of *Tnfsf11* and *Tnfrsf11b* (*n* = 4). Data represent mean ± SEM (error bars). *p < 0.05; **p < 0.01; ***p < 0.001; ****p < 0.0001 (one‐way ANOVA with Tukey's multiple comparisons test). D + Q, dasatinib + quercetin; IHC, Immunohistochemistry; SEM, standard error of the mean; ANOVA, analysis of variance; PTH, parathyroid hormone; OPN, osteopontin; P1NP, propeptide of type I collagen; *Tnfsf11*, tumor necrosis factor superfamily member 11; *Tnfrsf11b*, tumor necrosis factor superfamily member 1b; *p16INK4a*, cyclin‐dependent kinase inhibitor 2A.

Next, we investigated whether the combination of senolytic drugs, which selectively eliminate senescent cells, with PTH administration can eliminate senescent cells and prevent the deterioration of bone microstructure associated with PTH administration in aged mice. The senolytic drug combination of dasatinib and quercetin (D + Q) was co‐administered with PTH 10 times per week to aged mice over a 4‐week period (Figure 4A and Table 2). The frequency of PTH administration was determined based on the observed differences in trabecular and cortical bone morphology, as well as the accumulation of senescent cells between young and aged mice. Microstructural analysis using micro‐computed tomography (CT) revealed that the combination of PTH and D + Q reduced cortical porosity compared to PTH alone (Figure 4B,C). In contrast, no significant changes were observed in the trabecular bone and Ct.Th following the combination treatment (Figure 4D–H). Finite element analysis (FEA) revealed that co‐administration of D + Q with PTH increased spine failure load compared to PTH treatment alone (Figure S5C). Simultaneous administration of PTH and D + Q reduced the expression levels of *p16INK4a* and *SASP‐*related genes in the vertebrae compared to PTH administration alone (Figure 4I–N). IHC staining for p16INK4a and OPN indicated a decrease in the number of senescent osteoblast‐lineage‐enriched cells in the group undergoing combined PTH and D + Q treatment compared to that in the PTH alone group (Figure 5A,B). In the analysis of serum bone metabolism markers, co‐administration of D + Q did not alter P1NP levels but significantly decreased TRAcP5b levels (Figure 5C,D). The expression of tumor necrosis factor superfamily member 11 (*Tnfsf11*), whose transcript is a receptor activator of nuclear factor‐κB ligand (RANKL), was increased by PTH and exhibited a trend toward reduction with combined D + Q administration (*p* = 0.09). The expression of *Tnfrsf11b*, whose transcript is osteoprotegerin (OPG), was significantly upregulated with the combined PTH and D + Q treatment (Figure 5E,F). These findings were consistent with the results of OC.S/BS, as assessed by HE staining. Specifically, the increase in osteoclasts induced by PTH was suppressed by the co‐administration of D + Q (Figure S2D). Such results indicate that the accumulation of senescent cells induced by PTH administration, along with the activation of osteoclasts mediated by the SASP, contributes to the increase in cortical porosity observed in aged mice following PTH treatment.

**TABLE 2:** Dosing frequency and dosage of parathyroid hormone and senolytic drug administration.

Groups	Treatment (dosing)	Medication (dosing)
Control	Vehicle (10 times a week)	Vehicle (once a week)
PTH	PTH 200 μg/kg/week (10 times a week)	
PTH‐D + Q	PTH 200 μg/kg/week (10 times a week)	Dasatinib 5 mg/kg/day Quercetin 50 mg/kg/day (once a week)
D + Q	Vehicle (10 times a week)	

Abbreviations: D + Q, dasatinib + quercetin; PTH, parathyroid hormone.

### 2.7 Co‐Administration of Senolytics Attenuated Bone Loss After PTH Discontinuation

**FIGURE 6:**
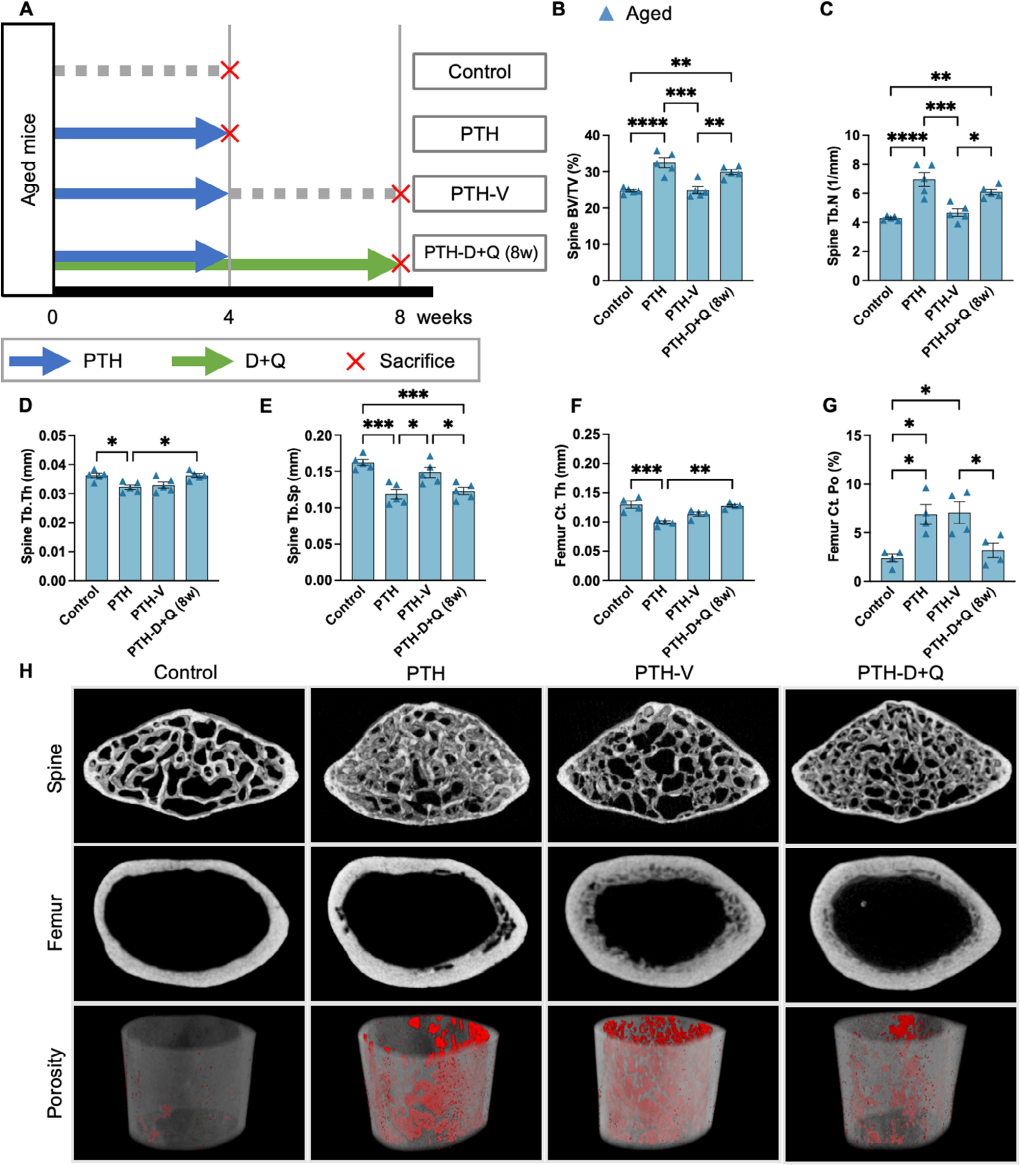
Senolytic treatment attenuated bone loss after discontinuation of parathyroid hormone treatment in aged mice. (A) Experimental timeline illustrating the PTH treatment (10 times a week) and subsequent discontinuation, with or without D + Q co‐treatment. (B–E) Quantitative analysis of trabecular bone parameters (BV/TV, Tb. N, Tb. Th, and Tb. Sp) (*n* = 5). (F–G) Analysis of cortical bone parameters (Ct. Th, Ct. Po) (*n* = 5). (H) Representative micro‐CT images of the lumbar spine and femur in aged mice. Cortical porosity is highlighted in red (below). Data represent mean ± SEM (error bars). *p < 0.05; **p < 0.01; ***p < 0.001; ****p < 0.0001 (one‐way ANOVA with Tukey's multiple comparisons test). PTH, parathyroid hormone; D + Q, dasatinib + quercetin; BV/TV, bone volume; Tb. N, trabecular number; Tb. Th, trabecular thickness; Tb. Sp, trabecular spacing; Ct. Th, cortical thickness; Ct. Po, cortical porosity; CT, computed tomography; SEM, standard error of the mean; ANOVA, analysis of variance.

Finally, we investigated whether the accumulation of senescent cells induced by PTH administration contributes to the rapid bone loss observed following the discontinuation of PTH treatment (Leder et al. 2009) and whether the clearance of senescent cells can mitigate bone loss caused by deterioration of the bone microstructure in trabecular and cortical bones. In aged mice, an increase in BV/TV was observed during the 4 weeks of PTH administration; however, the subsequent 4 weeks of vehicle treatment alone resulted in a rapid decline in BV/TV, returning to levels comparable to those of the control group (Figure 6A,B). Co‐administration of D + Q during the PTH treatment period and for 4 weeks after PTH withdrawal effectively maintained the increased BV/TV during the 4‐week PTH treatment and prevented the loss of BV/TV following discontinuation of PTH treatment (Figure 6B). Furthermore, D + Q administration mitigated the deterioration of both trabecular and cortical bone microstructural parameters, including BV/TV, Tb.N, Tb.Sp, and Ct.Po after the discontinuation of PTH treatment (Figure 6B–H). Although a mild improvement in BV/TV was observed following 8 weeks of D + Q treatment, no significant alterations were detected in other bone structural parameters (Figure S7B–G). Conversely, in young mice, BV/TV did not decrease with the 4 weeks of vehicle administration following the initial 4‐week PTH treatment (Figure S6A,B). No significant differences in bone structural parameters were observed between the vehicle and D + Q co‐treatment groups (Figure S7H–M). Similarly, other bone microstructural parameters in young mice did not deteriorate following PTH discontinuation, and D + Q administration did not induce significant changes in these parameters (Figure S6B–H). These findings suggest that rapid bone loss following the discontinuation of PTH administration in aged mice is at least partly driven by the accumulation of senescent osteoblast‐lineage‐enriched cells and that bone loss can be mitigated by preventing the accumulation of senescent cells.

## 3 Discussion

This study investigated the relationship between bone loss following PTH discontinuation and the impact of PTH administration on cellular senescence. PTH administration induced distinct changes in bone microstructural indices in aged and young mice. In aged mice, PTH treatment led to a decline in bone microstructural quality, characterized by cortical and trabecular thinning and increased cortical porosity, compared to young mice. Despite increased cortical porosity following high‐frequency PTH treatment, the failure load remained elevated. Given that previous studies have shown that trabecular bone contributes significantly to bone strength, this increase in failure load is likely attributable to the enhanced trabecular bone mass (Chiba et al. 2022). Notably, PTH treatment resulted in the accumulation of senescent cells in the bone tissues of aged mice. Furthermore, suppressing the accumulation of senescent cells using senolytic agents effectively mitigated both the deterioration of bone microstructure during PTH treatment and subsequent bone loss following PTH discontinuation in aged mice. These findings indicate that senescent cell accumulation contributes to the clinically significant issue of bone loss after PTH discontinuation. Thus, therapeutic strategies targeting senescent cells in the bone may not only prevent post‐treatment bone loss but also enhance the anabolic efficacy of PTH in older individuals.

Recent advances in osteoporosis drugs have enabled a rapid increase in BMD (Arceo‐Mendoza and Camacho 2021). However, without appropriate sequential therapy after discontinuation, the gained BMD decreases rapidly and can worsen even from baseline (Leder et al. 2009). PTH promotes the proliferation of pre‐osteoblasts (Yamamoto et al. 2016), and the localization of senescent cells in bone tissue following PTH administration suggests that, although the anabolic effects of PTH are predominant during its administration, senescent osteoblasts may contribute to rapid bone loss observed after the discontinuation of PTH treatment. In a previous animal model that specifically induced cellular senescence in osteoblasts, we observed cortical and trabecular bone thinning, which was ameliorated by the removal of senescent cells (Farr et al. 2017). Clinical studies have also demonstrated that daily PTH administration increases cortical porosity and reduces BMD (Chiba et al. 2022), consistent with our observations in an osteoblast senescence animal model.

In the present study, we found that young mice treated with high‐frequency PTH did not exhibit cortical bone porosity or reduced BMD. These findings indicate that the deterioration of bone microstructure and the accumulation of senescent cells induced by PTH administration are not solely attributable to the direct effects of PTH but also the cumulative impact of various cellular stresses. Compared with young mice, aged mice, which have already accumulated cellular stress, are more likely to become senescent due to PTH administration.

Importantly, our mechanical analyses further highlight that high‐frequency PTH exerts two opposing biological effects—enhancement of trabecular bone mass and deterioration of cortical bone structure. This duality explains the discrepancy observed between the density‐based FEA and the experimental three‐point bending tests (Figure S5E,F). Because FEA is highly sensitive to cortical geometry and density, but limited in its ability to model post‐yield behavior, micro‐porosity, and damage accumulation, it predominantly reflects the cortical deterioration induced by PTH (Hansen et al. 2013; Keaveny et al. 2008). In contrast, experimental bending tests capture whole‐bone failure mechanics and therefore represent both the trabecular augmentation and cortical degradation. Thus, these results indicate that the mechanical outcome of high‐frequency PTH treatment in aged mice is determined by the balance between these contrasting structural effects.

From a molecular biological perspective, we verified that PTH induced senescence in osteoblasts. mTORC1 activation promotes preosteoblast senescence through the regulation of sodium channels, and PTH binds to PTH1R in osteoblasts and preosteoblasts, increases Cyclic adenosine monophosphate (cAMP) production, and contributes to proliferation and survival via the PI3K/Akt and mitogen‐activated protein kinase kinase (MEK)/ERK pathways (Chen et al. 2022; Panwar et al. 2023; Yamamoto et al. 2007). Therefore, we examined the effects of PTH administration on mTORC1 and its downstream signaling. The activation of mTORC1, p‐S6K, and p‐4EBP1 was confirmed by PTH administration in mouse calvarial osteoblasts. mTORC1 is also involved in cell proliferation and survival (Cho et al. 2021; Panwar et al. 2023). Our findings suggest that PTH‐induced activation of the mTORC1 pathway did not increase senescent cells in young mice, where senescence‐related stress has not yet accumulated. In contrast, an expansion of senescent cells was observed exclusively in the bone tissues of aged mice, where replicative senescence and other forms of cellular stress had already accumulated. These findings suggest that PTH administration induced osteoblast senescence only in aged mice and that the accumulation of senescent cells formed a proinflammatory bone microenvironment.

In evaluating serum bone metabolism markers, the increase in the bone formation marker P1NP following PTH administration was the same in young and old mice; however, the bone resorption marker TRAcP5b showed higher values in aged mice than in young mice as the frequency of administration increased. In aged mice, D + Q suppressed PTH‐induced changes in *Tnfsf11* and *Tnfrsf11b*. Since senescent osteocytes can upregulate RANKL and downregulate OPG (Ben‐awadh et al. 2014; Kim et al. 2020; Silva and Bilezikian 2015; Wang et al. 2021), the discrepancy in bone formation in aged mice might be due to the accumulation of senescent osteoblasts and osteocytes in the bone microenvironment, which leads to osteoclast activation mediated by RANKL and the proinflammatory SASP (Farr and Khosla 2019; Li et al. 2023).

Combination treatment with D + Q targets and eliminates senescent cells (Farr et al. 2024; Farr et al. 2017; Raffaele and Vinciguerra 2022). In our study, D + Q treatment induced distinct changes in bone microarchitecture during the 4 weeks of PTH administration and the 4 weeks post‐PTH treatment. Specifically, the concurrent administration of D + Q during the 4‐week PTH treatment period improved cortical porosity but had no significant effects on other parameters of bone microarchitecture. In contrast, extending D + Q administration for an additional 4 weeks after discontinuation of PTH treatment improved all cortical and trabecular bone parameters and effectively prevented bone loss associated with PTH discontinuation. Although the effects of senolytic reagents were not complete, key parameters such as cortical porosity were restored to levels comparable to the control group, supporting a functional contribution of senescent cells to the post‐withdrawal phenotype. However, senescent cell accumulation may not be the sole cause of the rapid bone loss after PTH discontinuation. It is likely that multiple mechanisms, including changes in bone remodeling dynamics and hormonal signaling, are involved. Nevertheless, our findings strongly suggest that cellular senescence and SASP play critical roles in this process.

We hypothesize that the following two factors contributed to these outcomes. First, persistent cellular stress, such as replicative stress, and enhancement of the mTORC1 pathway induced by PTH may hinder the complete clearance of senescent cells by D + Q (Chen et al. 2022; Herr et al. 2024; Panwar et al. 2023). Second, D + Q administration improves both cortical and trabecular bone structures in aged mice; however, approximately 4 months of treatment are typically required to achieve this effect (Farr et al. 2023; Farr et al. 2017). Consequently, the improvement in bone quality was evident only after 8 weeks of combined PTH and D + Q treatment, including the 4 weeks following PTH discontinuation.

In this study, high‐frequency PTH administration in aged mice resulted in trabecular and cortical bone thinning and increased cortical porosity. These bone microstructural changes are consistent with those observed in older adults undergoing PTH treatment, as reported in previous clinical studies (Chiba et al. 2022; Takada et al. 2024; Zebaze et al. 2017). These findings suggest that the therapeutic efficacy of PTH is diminished in aged bones, which are characterized by the accumulation of senescent cells. Furthermore, rapid bone loss following discontinuation of PTH treatment has also been observed exclusively in aged mice. Importantly, post‐treatment bone loss was significantly mitigated by the removal of senescent cells with the combined administration of D + Q.

Although the effects of senolytic treatment were not comprehensive, key parameters—particularly cortical porosity, which was markedly increased following PTH withdrawal—were restored to levels comparable to those of the control group. This supports a functional role of senescent cells in mediating the adverse bone phenotype observed after PTH discontinuation. However, senescent cell accumulation may not be the sole driver of this rapid bone loss. It is more plausible that multiple interrelated mechanisms, including altered bone remodeling dynamics and disrupted hormonal signaling, contribute to the observed changes. Nonetheless, our findings provide compelling evidence that cellular senescence and associated SASP factors constitute critical components of the bone loss observed after PTH discontinuation.

Therefore, therapeutic strategies targeting senescent cells in conjunction with PTH treatment may represent a promising approach to enhance the efficacy and sustainability of PTH therapy, particularly in older individuals who account for the majority of patients with osteoporosis.

This study has limitations. First, we did not examine senescent cells other than osteoblast‐lineage‐enriched cells in the bone microenvironment following PTH administration. However, based on our findings, we believe that osteoblasts play a major role in the accumulation of senescent cells and the SASP caused by PTH (Figures S8A,B, S9A,B). Additional research is needed to clarify the involvement of other bone cells (bone marrow stromal cells, osteoclasts, and osteocytes). Moreover, although OPN was used as a marker for osteoblasts and p16INK4a as a marker for senescent cells, future studies should incorporate multiple markers and detection approaches to ensure more comprehensive and accurate identification. Previous studies have often focused on senescent osteocytes, but they could not be confirmed in this study. IHC involved fixation and demineralization, and osteocytes within lacunae were anticipated to stain poorly. Thus, further investigation into the involvement of osteocytes is warranted. The second limitation of this study is that senolytic agents were administered systemically, and thus, the possibility of off‐target effects on non‐osteoblastic cells within the bone microenvironment (e.g., osteocytes, stromal cells, or immune cells) or in peripheral tissues cannot be excluded. While our histological and molecular analyses suggest predominant effects on osteoblast‐lineage‐enriched cells, future studies employing cell‐type‐specific delivery systems or genetic approaches will be necessary to precisely define the cellular targets of senolytic therapy and limit systemic exposure. Third, the duration of both PTH administration and senolytic treatment was relatively short, which may have affected the magnitude of the changes observed in the micro‐CT after senescent cell removal. Fourth, only male mice were used in the in vivo experiments. Given that sex‐specific differences can influence bone metabolism and regenerative responses, the findings should be interpreted with caution when extrapolating to female animals. Further studies incorporating both sexes will be necessary to validate the generalizability of the current results. Fifth is the absence of dynamic histomorphometric data, such as mineral apposition rate (MAR) and bone formation rate (BFR), which are typically assessed using calcein double labeling. Instead, static histological evaluations were performed using HE‐stained sections to quantify bone formation and resorption surfaces. Although this approach provides meaningful information about bone remodeling status, it does not allow for dynamic assessment of bone turnover. Future studies employing fluorochrome labeling will be necessary to characterize bone formation kinetics comprehensively. Although we observed concurrent mTORC1 activation and increased expression of senescence markers following PTH treatment, we did not directly test whether mTORC1 activation is responsible for driving osteoblast senescence. Future studies employing mTORC1 inhibition would be necessary to establish a causal relationship. It should be noted that although our intermittent PTH administration protocols (10 times/week and 5 times/week) were designed to functionally model daily and weekly PTH dosing in humans, respectively, they do not perfectly reproduce the pharmacokinetics or bone remodeling dynamics associated with clinical PTH administration and withdrawal. This is partly because bone turnover in rodents is two to three times higher than in humans, requiring more frequent dosing to achieve comparable anabolic or catabolic effects. As such, caution is warranted when extrapolating these findings directly to human clinical scenarios.

Nonetheless, our findings demonstrate the significant beneficial effects of an 8‐week senolytic treatment on both the cortical and trabecular bones. Further investigations are needed to determine whether longer durations of PTH and senolytic treatment can result in more pronounced changes.

In conclusion, this study demonstrated that PTH administration promoted the accumulation of senescent cells in the bone microenvironment, contributing to the deterioration of bone microarchitecture during PTH treatment and the rapid decline in bone mass following PTH discontinuation. These findings suggest that the efficacy of PTH therapy, a cornerstone of osteoporosis treatment, may be enhanced by therapeutic interventions targeting senescent cells.

## 4 Methods

### 4.1 Study Design

#### 4.1.1 PTH Dose and Frequency of Administration

First, we examined the effects of the frequency of PTH administration on bone mass and senescent cell accumulation in young and old mice. Based on the difference in bone remodeling between humans and rodents (Yamamoto et al. 2016), once or twice‐daily subcutaneous administration was performed for 4 weeks (the total dose of PTH was standardized to 40 μg/kg/5 days/week). The control group was administered vehicle (0.9% saline). After 4 weeks of treatment, the spine, femur, and tibiae were extracted for histological assessment and statistical analyses using quantitative reverse transcription PCR (RT‐qPCR). PTH was provided by Asahi Kasei Pharma Co. Ltd. (Tokyo, Japan).

#### 4.1.2 Administration of Senolytic Drugs

Mice were randomly assigned to receive either weekly oral administration with dasatinib (5 mg/kg, TCI, Tokyo, Japan) and quercetin (50 mg/kg, Fluorochem Ltd., Hadfield, Derbyshire, UK) combined (D + Q) or vehicle for 4 or 8 weeks, alongside twice‐daily administration of PTH (40 μg/kg, 5 days per week). D + Q, previously identified as senolytic agents, were administered weekly to minimize off‐target effects and efficacy in clearing senescent cells (Novais et al. 2021; Tappy et al. 2024). D + Q was dissolved in 10% polyethylene glycol 400 (PEG400; 100 μL) and delivered via oral gavage. The control group received an equivalent volume of vehicle (10% PEG400).

### 4.2 Animals

Three‐month‐old (young) and 18‐month‐old (aged) C57BL/6J male mice were obtained from Charles River Laboratories Japan Inc. (Kanagawa, Japan). The mice were maintained on a normal chow diet with a 12‐h light/dark cycle.

### 4.3 Micro‐CT Analysis

A high‐resolution micro‐CT scan (Skyscan 1272, Bruker, Billerica, MA) was used at a resolution of 5 μm per voxel. Parameters, including bone volume, tissue volume, trabecular and cortical thickness, trabecular separation, cortical porosity (Harrison et al. 2020), and mineral density, were analyzed using CTAn software (Bruker). Mineral density calibration was performed using a bone calibration phantom. Three‐dimensional reconstructed images were obtained using CTvox software (Bruker). The analysis area of the vertebrae was from a point 0.3–0.6 mm proximal to the distal growth plate, and the area of the femur was 6–8 mm proximal to the distal growth plate. Briefly, cortical bone analysis was performed using a region of interest defined as a 2‐mm segment starting 6 mm proximal to the distal femoral growth plate (Piemontese et al. 2017).

### 4.4 RT‐qPCR

Total RNA was isolated using the TRIzol reagent (Thermo Fisher Scientific, Waltham, MA). Bone (vertebrae, femurs, and tibiae) preprocessing for in vivo assays was performed, as previously described (Farr et al. 2016; Stern et al. 2012). Briefly, we used femoral, tibial, and lumbar bones from mice for RNA isolation. Bones were minced into small pieces and subjected to two sequential 30‐min collagenase digestions (Sigma‐Aldrich, St. Louis, MO). Total RNA was extracted from the remaining bone pieces. These digestions contain osteoblast‐ and osteocyte‐enriched cells (Farr et al. 2016; Stern et al. 2012). Complementary DNA was synthesized using ReverTra Ace (TOYOBO, Osaka, Japan) and used as a template for qPCR with FAST SYBR Green Master Mix (Thermo Fisher Scientific). Target mRNA levels were normalized to the reference gene expression. The median threshold cycle was compared with that of the control sample, and the fold difference between the reference and target genes was calculated. *Glyceraldehyde‐3‐phosphate dehydrogenase* (*GAPDH*) was used as an internal control for in vivo assays. The primer sequences used for RT‐qPCR are listed in Table S1.

### 4.5 Biomechanical Testing

A three‐point bending test was conducted using a mechanical testing machine (Instron‐5565; Instron, Norwood, MA) to investigate the biomechanical strength of the femoral bone. The femurs were placed on a pedestal with the anterior side facing downward. A constant vertical compression load (3 mm/min) was applied to the midpoint of each specimen until failure. Load and displacement data were acquired every 10 ms. From the load–displacement curve, the ultimate load at failure (N; maximum load that the bone could bear) was determined.

### 4.6 FEA of Mouse Lumbar Vertebrae and Femora

FEA was performed to estimate the mechanical properties of mouse lumbar vertebrae and femora based on micro‐CT imaging data. Lumbar vertebrae (L4) and femora were scanned using high‐resolution micro‐CT (Skyscan 1272, Bruker; voxel size: 5 μm). The acquired 3D datasets were segmented using a global thresholding method to distinguish mineralized bone from soft tissue. Image processing, segmentation, and conversion to finite element meshes were performed using TRI/3D‐BON‐FCS64 Bone Morphometry Software (RATOC System Engineering Co. Ltd., Tokyo, Japan). For vertebral analysis, a linear elastic and isotropic material model was used. Uniaxial compression was applied to the superior endplate while the inferior endplate was fully constrained. Failure load and energy to failure were computed. For femoral analysis, FE models were generated under three‐point bending test conditions, consistent with the in vitro setup. To reproduce the mechanical boundary conditions accurately, the femur was fixed at two support points located 4 mm proximal and 4 mm distal to the mid‐diaphysis. A downward displacement was then applied at the central loading point to simulate bending. Estimated failure load and failure energy were calculated and compared across groups.

### 4.7 Histological Analysis of Bone Remodeling Surfaces

Quantitative assessment of bone formation and resorption surfaces was performed on HE‐stained decalcified sections. Bone‐forming surfaces were identified based on the presence of cuboidal osteoblasts lining the trabeculae, while bone‐resorbing surfaces were defined by multinucleated osteoclasts in resorption lacunae. The proportions of these surfaces were quantified by manual counting performed by a bone histomorphometry specialist blinded to the experimental groups.

### 4.8 Osteoblast Isolation From Neonatal Murine Calvaria

Primary osteoblasts were obtained from the calvaria of neonatal mice (postnatal age 2–3 days) (Jonason and O'Keefe 2014). Briefly, calvaria were minced and digested four times in alpha‐Minimum Essential Medium (α‐MEM) (Nacali Tesque, Kyoto, Japan), 0.176% NaHCO_3_, and 0.008% collagenase type II (ThermoFisher Scientific) at 37°C for 15 min. Supernatants from the third and fourth digestions were collected. After centrifugation, cell suspensions were incubated in 10‐cm dishes with α‐MEM, 10% Fetal bovine serum (FBS), and 1% penicillin–streptomycin at 37°C with 20% O_2_ and 5% CO_2_. After 1 week, confluent cells (passage 0) were used for experiments.

### 4.9 Western Blot Analysis

The effects of PTH on osteoblast proliferation and gene expression were examined in vitro. Murine calvarial osteoblasts were seeded in six‐well plates at a density of 1.0 × 10^5^ cells per well. Cells were maintained in α‐MEM, supplemented with 10% FBS and 1% penicillin/streptomycin solution. Cells were incubated at 37°C in humidified incubators with 5% CO_2_. The cells were treated with 100 nM PTH for 24 h. After treatment, the cells were harvested for protein isolation. Protein concentrations were measured using bicinchoninic acid (Thermo Fisher Scientific), according to the manufacturer's protocol. The cell lysates were separated on 4%–12% Bis‐Tris gels (Life Technologies, Carlsbad, CA) and transferred to polyvinylidene difluoride membranes (Nippon Genetics, Tokyo, Japan), which were then incubated in Tris‐buffered saline containing 5% skim milk and Tween 20 (TBS‐T) at 22°C. The blocked membranes were incubated with primary antibodies in Can Get Signal Solution 1 (Toyobo) at 4°C overnight, after which they were incubated with secondary antibodies in Can Get Signal Solution 2 (Toyobo) at room temperature for 1 h. After washing with TBS‐T, the immunoreactive bands were visualized using the ECL Plus Western Blotting Detection System kit (Thermo Fisher Scientific) and ChemiDOC Touch (Bio‐Rad, Hercules, CA). The following antibodies were used for western blotting at the indicated dilutions: rabbit monoclonal anti‐4E‐BP1 (#9644, 1:1000, Cell Signaling Technology), rabbit monoclonal anti‐phospho‐4E‐BP1 (#2855, 1:1000, Cell Signaling Technology), rabbit monoclonal anti‐p70 S6 kinase (#9202, 1:1000, Cell Signaling Technology), rabbit monoclonal anti‐phospho‐p70 S6 kinase (#9234, 1:1000, Cell Signaling Technology), rabbit monoclonal anti‐p44/42 MAPK (Erk1/2) (#4695, 1:1000, Cell Signaling Technology), rabbit monoclonal anti‐phospho‐p44/42 MAPK (Erk1/2) (#4370, 1:1000, Cell Signaling Technology), rabbit monoclonal anti‐PI3 Kinase p110α (#4249, 1:1000, Cell Signaling Technology), and rabbit polyclonal anti‐β‐Actin (#4967, 1:1000, Cell Signaling Technology).

### 4.10 Histological Analysis

The excised samples were fixed in 10% neutral‐buffered formalin, decalcified with ethylenediaminetetraacetic acid, embedded in paraffin wax, and cut to a 3.5‐mm thickness. Bone sections were deparaffinized and dehydrated for immunostaining. The antigens were activated in 10 mM citrate buffer at 80°C for 30 min. After quenching the endogenous peroxidase activity with methanol containing 3% H_2_O_2_ for 10 min, the sections were blocked with Blocking One (Nacalai Tesque) for 20 min at room temperature. Sections were then incubated with a primary antibody overnight at 4°C, followed by incubation with a horseradish peroxidase‐labeled secondary antibody for 1 h. Finally, the labeled sections were stained with Histofine Simple Stain Mouse MAX PO (Nichirei Bioscience, Tokyo, Japan) and counterstained with hematoxylin.

For immunofluorescence, the bone sections were deparaffinized, rehydrated, permeabilized for 30 min with 0.3% Triton X‐100 in PBS, and blocked with Blocking One (Nacalai Tesque) at room temperature for 20 min. Blocked sections were incubated with primary antibodies overnight at 4°C. After incubation with the primary antibody, the sections were washed with PBS and incubated with the appropriate Alexa Fluor‐conjugated secondary antibodies (1:1000, Thermo Fisher Scientific) diluted in PBS for 1 h at room temperature. Sections were washed with PBS before mounting with ProLong Diamond Antifade Mountant with 4′,6‐diamidino‐2‐phenylindole (DAPI) (Thermo Fisher Scientific). The following primary antibodies were used: rabbit monoclonal anti‐Cyclin Dependent Kinase Inhibitor 2A (CDKN2A)/p16INK4a (ab211542, 1:250, Abcam, Cambridge, UK), rabbit monoclonal anti‐phospho‐S6 ribosomal protein (Ser240/244) (#5364, 1:500, Cell Signaling Technology), rabbit monoclonal anti‐phospho‐4EBP1 (1:800), mouse Leptin R antibody (AF497, 1:250, R&D Systems), mouse Dentin Matrix Protein 1 (DMP‐1) antibody (AF4386, 1:250, R&D Systems), Runt‐related Transcription Factor 2 (RUNX2) rabbit monoclonal antibody (#12556, 1:500, Cell Signaling Technology), anti‐osteocalcin antibody (ab309521, 1:500, Abcam), anti‐Ki67 antibody (ab15580, 1:500, Abcam), mouse Interleukin‐1 alpha (IL‐1α) antibody (AF‐400‐NA, 1:250, R&D Systems), and goat anti‐osteopontin antibody (AF808, 1:100, R&D Systems, Minneapolis, MN). Secondary antibodies used were donkey anti‐rabbit IgG conjugated to AF 594 (A21207, Invitrogen, Carlsbad, CA) and donkey anti‐goat immunoglobulin G (Zhu et al. 2015) conjugated to AF 488 (ab150129, Abcam). BZ‐X800 (Keyence, Osaka, Japan) was used to observe and capture the fluorescence images. The number of CDKN2A/p16INK4a and osteopontin‐positive cells in five randomly selected images of 270 × 360 μm in size from each section was counted automatically.

### 4.11 Evaluation of Serum Bone Metabolism Markers

Serum was collected from anesthetized mice via cardiac puncture and stored at −80°C for biochemical assays. P1NP (ng/mL) and TRAcP 5b (U/L) were quantified using a Rat/Mouse P1NP enzyme immunoassay kit (Immunodiagnostic Systems, Boldon, UK) and Mouse TRAcP 5b ELISA kit, respectively.

### 4.12 Statistical Analysis

Statistical analyses were performed using GraphPad Prism version 10.2.3 (GraphPad Software, San Diego, CA). All data are presented as mean ± standard error of the mean (SEM). One‐way and two‐way analysis of variance (ANOVA) were used to determine the statistical significance, as indicated in the legends. Statistical significance was set at *p* < 0.05. The sample sizes for each experiment are described in the corresponding figure legends.

### 4.13 Study Approval

All mice were cared for in strict compliance with and approval from the Institutional Animal Committee of our institution (approval number: 04‐082‐003) and restrictedly followed ARRIVE (Animal Research: Reporting of In Vivo Experiments) guidelines and the National Institutes of Health Guide for the Care and Use of Laboratory Animals.

## Author Contributions

M.B. performed the majority of the experiments and analyses and wrote the manuscript. Y.U. wrote the manuscript and supervised the experiments. H.H. performed experiments and analyses. M.I. performed experiments and analyses. T.K. performed experiments and analyses. T.F. performed experiments and analyses. D.T. performed experiments and analyses. Y.K. performed experiments and analyses. M.F. supervised the study. T.F. supervised the study. S.O. supervised the study and edited the manuscript. S.O. supervised the study. T.K. conceptualized and directed the study and wrote the manuscript.

## Funding

This work was supported by the Japan Society for the Promotion of Science, 20K09479, 23K15689 and by a collaborative research grant from Asahi Kasei Paharma Corporation.

## Ethics Statement

All mice were cared for in strict compliance with and approval from the Institutional Animal Committee of our institution (approval number: 04‐082‐003) and restrictedly followed ARRIVE guidelines and the National Institutes of Health Guide for the Care and Use of Laboratory Animals.

## Conflicts of Interest

The authors declare no conflicts of interest.

## Supporting information


**Figure S1:** Effects of parathyroid hormone treatment frequency on bone morphology of the femur in young and aged mice. (A) Schematic illustration of the experimental design. (B) Representative micro‐computed tomography (μCT) analysis of the femur of young and aged mice treated with PTH and vehicle (*n* = 4 mice/treatment). Quantitative analysis of trabecular bone volume (BV/TV) (C), trabecular thickness (Tb. Th) (D), trabecular number (Tb. N) (E), and trabecular spacing (Tb. Sp) (F), in femur. (G) Immunohistochemistry (IHC) for p16INK4a in the femur (see arrows [in below], scale bars, 500 μm; 50 μm in below). Data represent mean ± SEM (error bars). *p < 0.05; **p < 0.01; ***p < 0.001 (independent samples *t*‐test or Wilcoxon rank‐sum test, as appropriate).

**Figure S2:** Histological analysis of bone remodeling surfaces of the spine in young and aged mice. (A) Representative HE‐stained sections from each group. Blue arrows indicate osteoblasts, and yellow arrows indicate osteoclasts. (B) Quantification of osteoblast surface per bone surface (Ob.S/BS). (C) Quantification of osteoclast surface per bone surface (Oc.S/BS). (D) Oc.S/BS values in aged mice only. HE, hematoxylin and eosin; IHC, immunohistochemistry; PTH, parathyroid hormone; D + Q, dasatinib + quercetin.

**Figure S3:** Assessment of cellular senescence in osteoblasts. (A) IHC for Osteopontin (OPN), Osteocalcin (OC), Runt‐related transcription factor 2 (RUNX2), p16INK4a, and p21 in the lumbar spine of aged mice (scale bars, 50 μm). (B) RT‐qPCR analysis of *p21* mRNA expression levels in osteoblast and osteocyte‐enriched cells derived from the bones of aged mice (*n* = 4 mice/treatment). (C) IHC for Ki‐67 in the lumbar spine of young and aged mice (scale bars, 50 μm). IHC, immunohistochemistry; RT‐qPCR, reverse transcription quantitative polymerase chain reaction.

**Figure S4:** Parathyroid hormone–induced accumulation of senescent cells in young mice. RT‐qPCR analysis of mRNA expression levels of *p16INK4a* (A) and SASP factors (*IL6*, *IL1α*, *IL1β*, *Mmp3*, *MMP13*, *Ccl5*) (B–G) in osteoblast and osteocyte‐enriched cells derived from the bones of aged mice (*n* = 4 mice/treatment). (H) IHC for p16INK4a in the lumbar spine of aged mice (see arrows, scale bars, 500 μm; 50 μm on right). Data represent mean ± SEM (error bars). SEM, standard error of the mean.

**Figure S5:** Finite element analysis of aged mice. (A–D) Finite element analysis (FEA) result for failure load and failure energy of the spine (*n* = 3). Data represent mean ± SEM (error bars). *p < 0.05; **p < 0.01 (A, B: independent samples *t*‐test). SEM, standard error of the mean; PTH, D + Q, dasatinib + quercetin.

**Figure S6:** Discontinuation of parathyroid hormone treatment or senolytic treatment caused no significant changes in the bone of young mice. (A) Experimental timeline illustrating the PTH treatment (10 times a week) and subsequent discontinuation, with or without D + Q co‐treatment. (B–E) Quantitative analysis of trabecular bone parameters (BV/TV, Tb. N, Tb. Th, and Tb. Sp) (*n* = 5). (F–G) Analysis of cortical bone parameters (Ct. Th, Ct. Po) (*n* = 5). (H) Representative micro‐CT images of the lumbar spine and femur in aged mice. Cortical porosity is highlighted in red (below). Data represent mean ± SEM (error bars). *p < 0.05; **p < 0.01; ***p < 0.001; ****p < 0.0001 (one‐way ANOVA with Tukey's multiple comparisons test). PTH, parathyroid hormone; D + Q, dasatinib + quercetin; BV/TV, bone volume; Tb. N, trabecular number; Tb. Th, trabecular thickness; Tb. Sp, trabecular spacing; Ct. Th, cortical thickness; Ct. Po, cortical porosity; CT, computed tomography; SEM, standard error of the mean; ANOVA, analysis of variance.

**Figure S7:** Effects of 8 weeks of senolytic treatment (dasatinib + quercetin) on bone morphology. (A) Experimental design for D + Q treatment in young and aged mice. (B–E) Quantitative analysis of trabecular bone parameters (BV/TV, Tb. N, Tb. Sp, Tb. Th) in aged mice (*n* = 5 mice/treatment). Analysis of (F) cortical thickness (Ct. Th) and (G) cortical porosis (Ct. Po) in aged mice. (H–K) Quantitative analysis of trabecular bone parameters (BV/TV, Tb. N, Tb. Sp, Tb. Th) in young mice (*n* = 5 mice/treatment). Analysis of (L) cortical thickness (Ct. Th) and (M) cortical porosis (Ct. Po) in young mice. D + Q, dasatinib + quercetin; BV/TV, bone volume; Tb. N, trabecular number; Tb. Th, trabecular thickness; Tb. Sp, trabecular spacing. *p < 0.05; (independent samples *t*‐test).

**Figure S8:** Immunofluorescent co‐localization of leptin receptor and p16INK4a in bone tissue. (A) Double immunofluorescence staining for p16INK4a (red) and leptin receptor (Leptin R) in the lumbar spine of aged mice (Scale bars, 50 μm). (B) percentage of double‐positive cells (p16+/Leptin R+) per p16 positive cells (*n* = 4). *p < 0.05 (independent samples *t*‐test). PTH, parathyroid hormone.

**Figure S9:** Immunofluorescent co‐localization of Dmp1 and IL1α with p16INK4a. (A) Double immunofluorescence staining for p16INK4a (red) and Dentin matrix protein 1 (Dmp1) in the lumbar spine of aged mice (Scale bars, 50 μm). (B) Double immunofluorescence staining for p16INK4a (red) and Interleukin‐1 alpha (IL1α) in the lumbar spine of aged mice (Scale bars, 50 μm). DAPI, 4′,6‐diamidino‐2‐phenylindole; PTH, parathyroid hormone.

**Table S1:** Primers for RT‐qPCR.

## Data Availability

The datasets generated and analyzed in the current study are available from the corresponding author upon reasonable request.
