# Supplementary figures and images for: Senescent Cells Involved in Deterioration of Bone Microstructure by High‐Frequency Parathyroid Hormone 1–34 Administration and Bone Loss
Source: Aging Cell. 2025 Dec 23;25(1):e70331. doi: 10.1111/acel.70331 (PMC12723732; doi:10.1111/acel.70331)

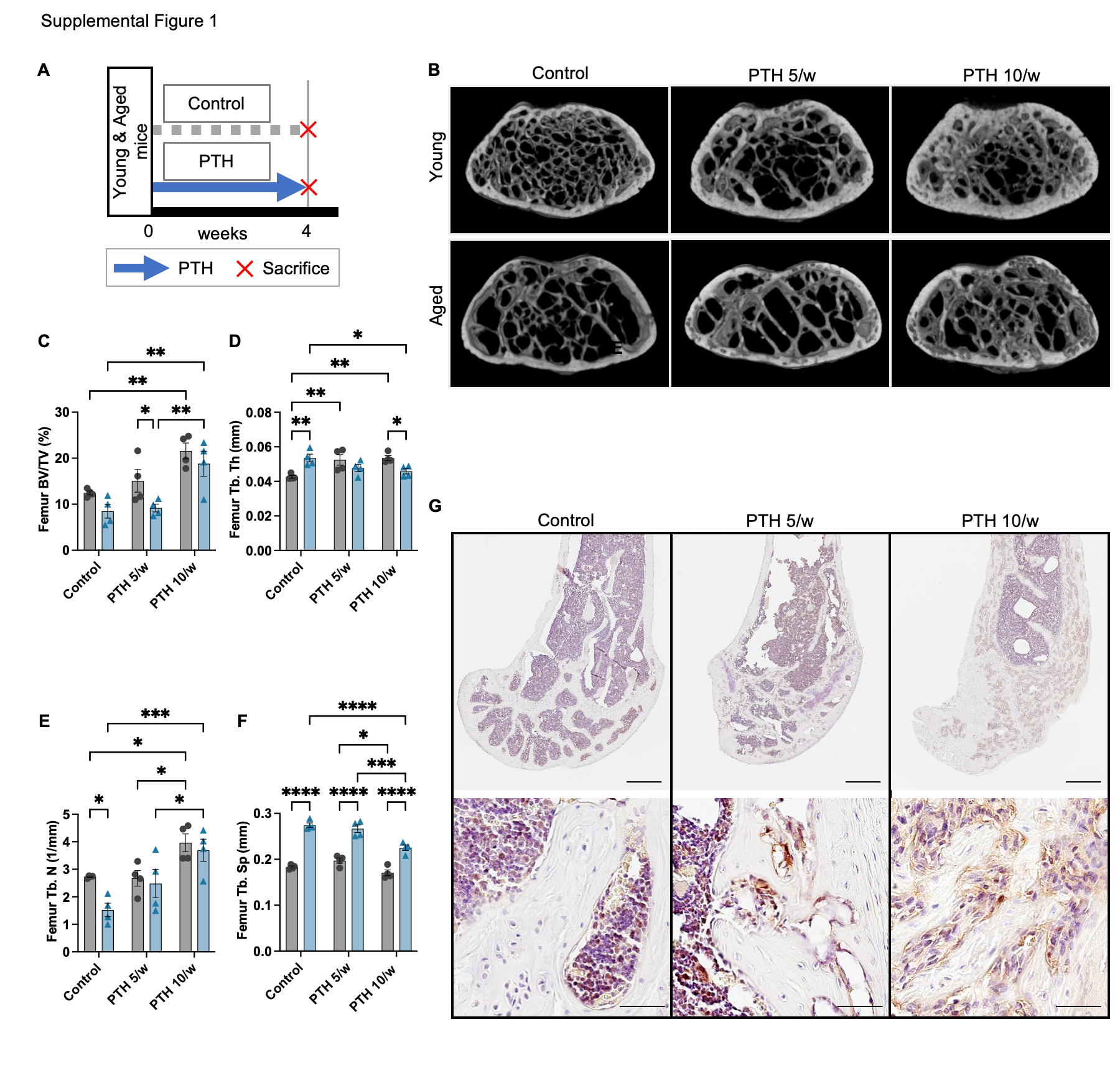

Supplement: Supplementary file 1 — Figure S1: Effects of parathyroid hormone treatment frequency on bone morphology of the femur in young and aged mice. (A) Schematic illustration of the experimental design. (B) Representative micro‐computed tomography (μCT) analysis of the femur of young and aged mice treated with PTH and vehicle (n = 4 mice/treatment). Quantitative analysis of trabecular bone volume (BV/TV) (C), trabecular thickness (Tb. Th) (D), trabecular number (Tb. N) (E), and trabecular spacing (Tb. Sp) (F), in femur. (G) Immunohistochemistry (IHC) for p16INK4a in the femur (see arrows [in below], scale bars, 500 μm; 50 μm in below). Data represent mean ± SEM (error bars). *p < 0.05; **p < 0.01; ***p < 0.001 (independent samples t‐test or Wilcoxon rank‐sum test, as appropriate). [file ACEL-25-e70331-s003.tiff]

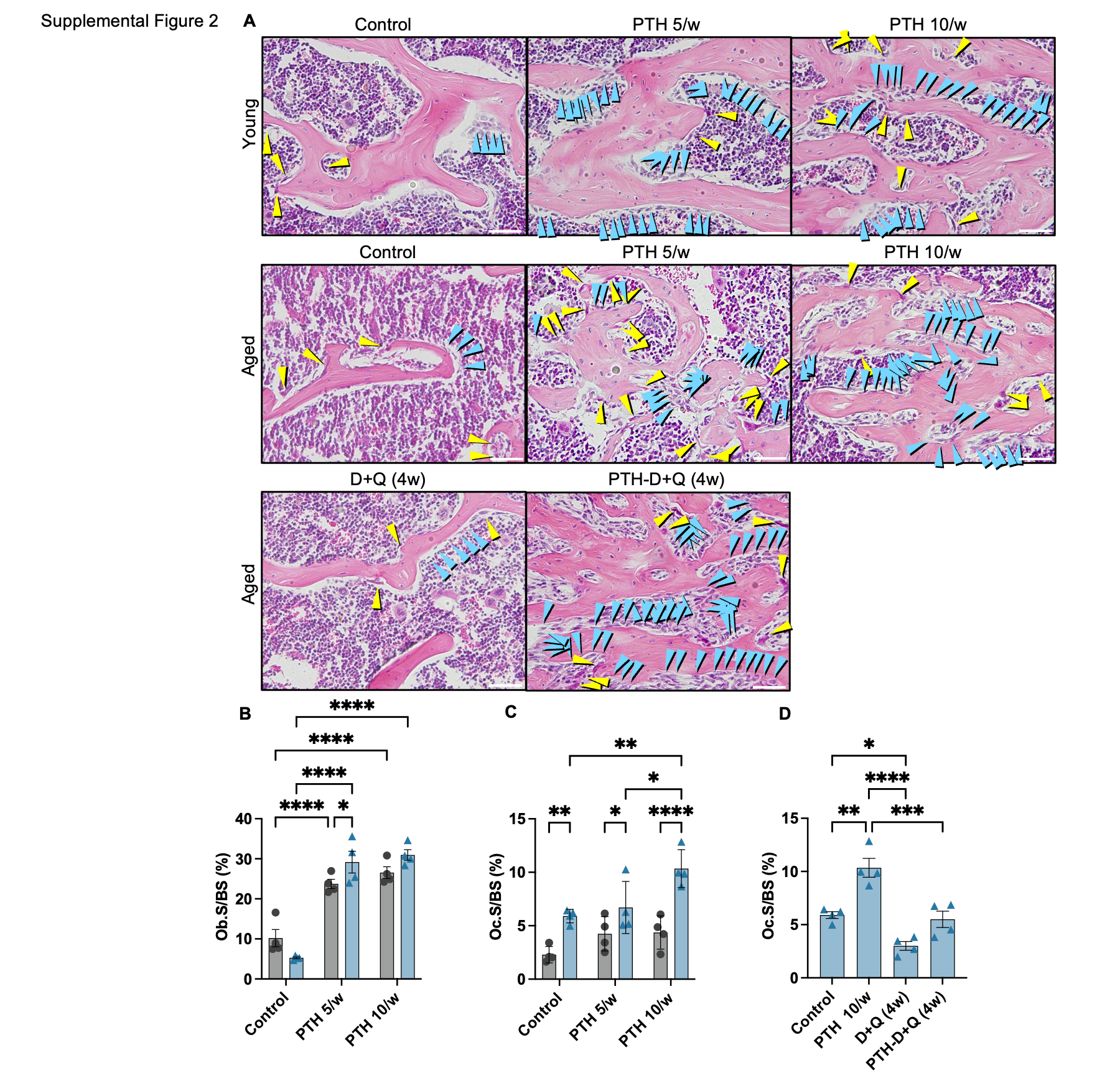

Supplement: Supplementary file 2 — Figure S2: Histological analysis of bone remodeling surfaces of the spine in young and aged mice. (A) Representative HE‐stained sections from each group. Blue arrows indicate osteoblasts, and yellow arrows indicate osteoclasts. (B) Quantification of osteoblast surface per bone surface (Ob.S/BS). (C) Quantification of osteoclast surface per bone surface (Oc.S/BS). (D) Oc.S/BS values in aged mice only. HE, hematoxylin and eosin; IHC, immunohistochemistry; PTH, parathyroid hormone; D + Q, dasatinib + quercetin. [file ACEL-25-e70331-s007.tiff]

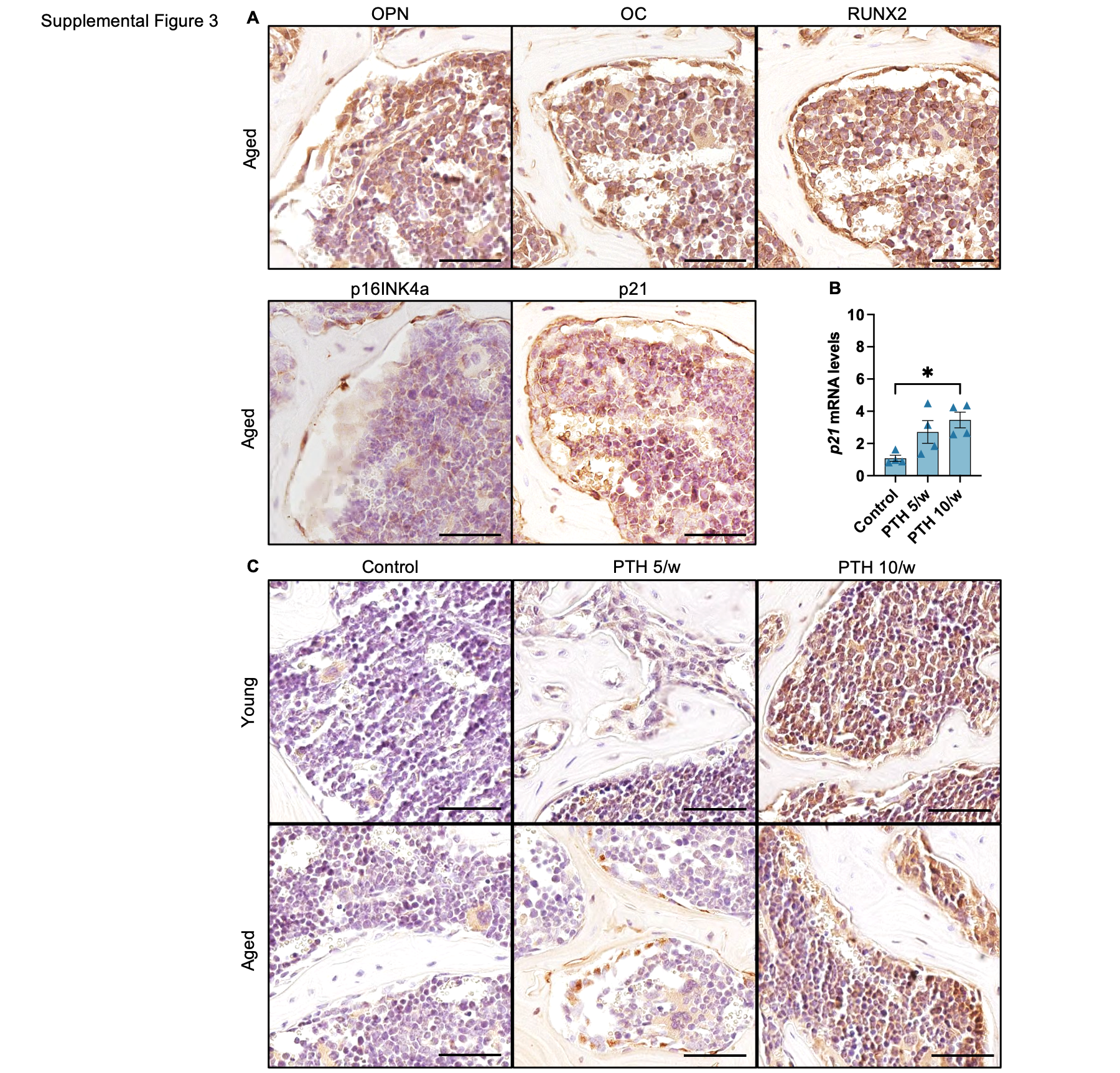

Supplement: Supplementary file 3 — Figure S3: Assessment of cellular senescence in osteoblasts. (A) IHC for Osteopontin (OPN), Osteocalcin (OC), Runt‐related transcription factor 2 (RUNX2), p16INK4a, and p21 in the lumbar spine of aged mice (scale bars, 50 μm). (B) RT‐qPCR analysis of p21 mRNA expression levels in osteoblast and osteocyte‐enriched cells derived from the bones of aged mice (n = 4 mice/treatment). (C) IHC for Ki‐67 in the lumbar spine of young and aged mice (scale bars, 50 μm). IHC, immunohistochemistry; RT‐qPCR, reverse transcription quantitative polymerase chain reaction. [file ACEL-25-e70331-s006.tiff]

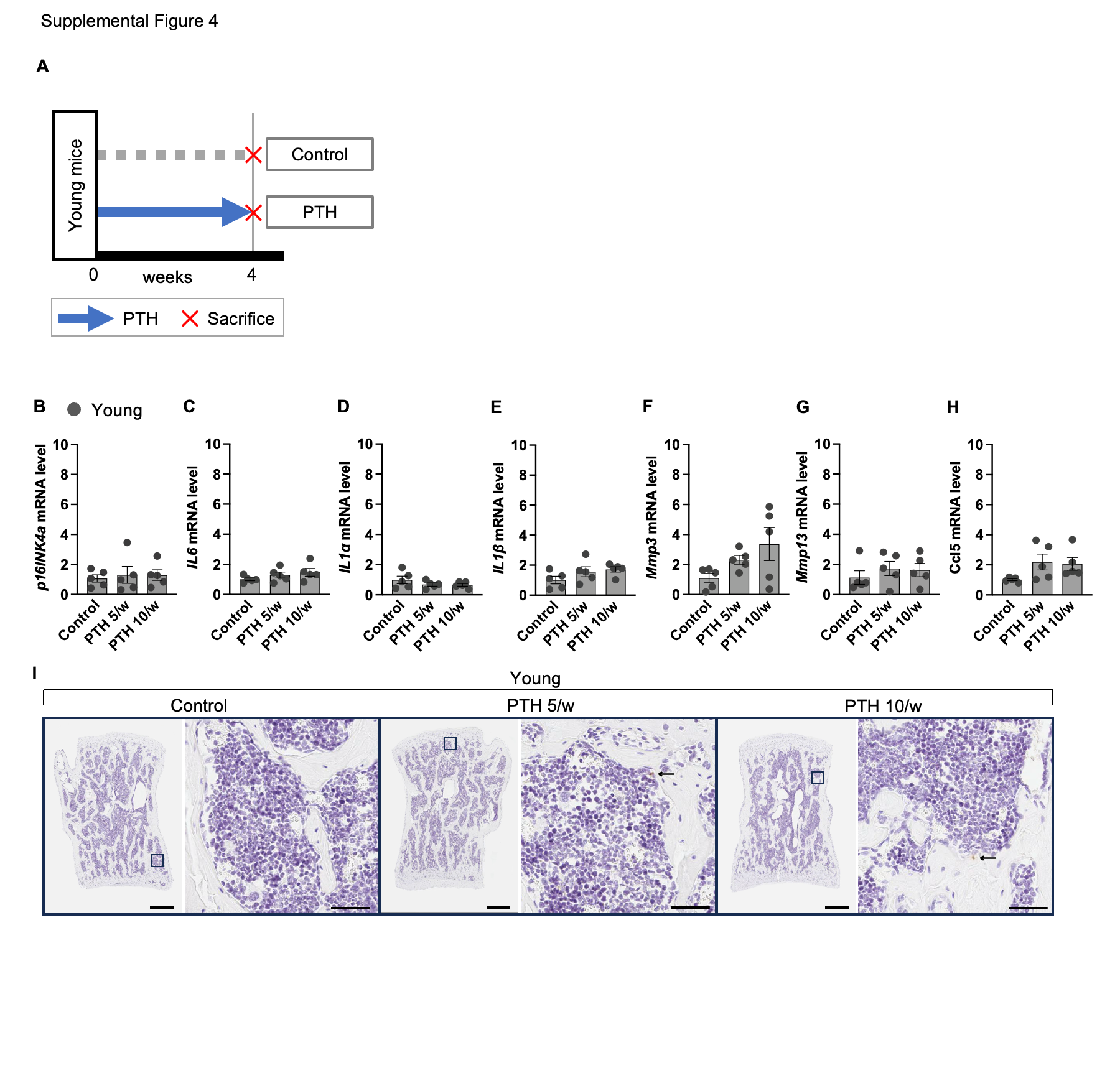

Supplement: Supplementary file 4 — Figure S4: Parathyroid hormone–induced accumulation of senescent cells in young mice. RT‐qPCR analysis of mRNA expression levels of p16INK4a (A) and SASP factors (IL6, IL1α, IL1β, Mmp3, MMP13, Ccl5) (B–G) in osteoblast and osteocyte‐enriched cells derived from the bones of aged mice (n = 4 mice/treatment). (H) IHC for p16INK4a in the lumbar spine of aged mice (see arrows, scale bars, 500 μm; 50 μm on right). Data represent mean ± SEM (error bars). SEM, standard error of the mean. [file ACEL-25-e70331-s005.tiff]

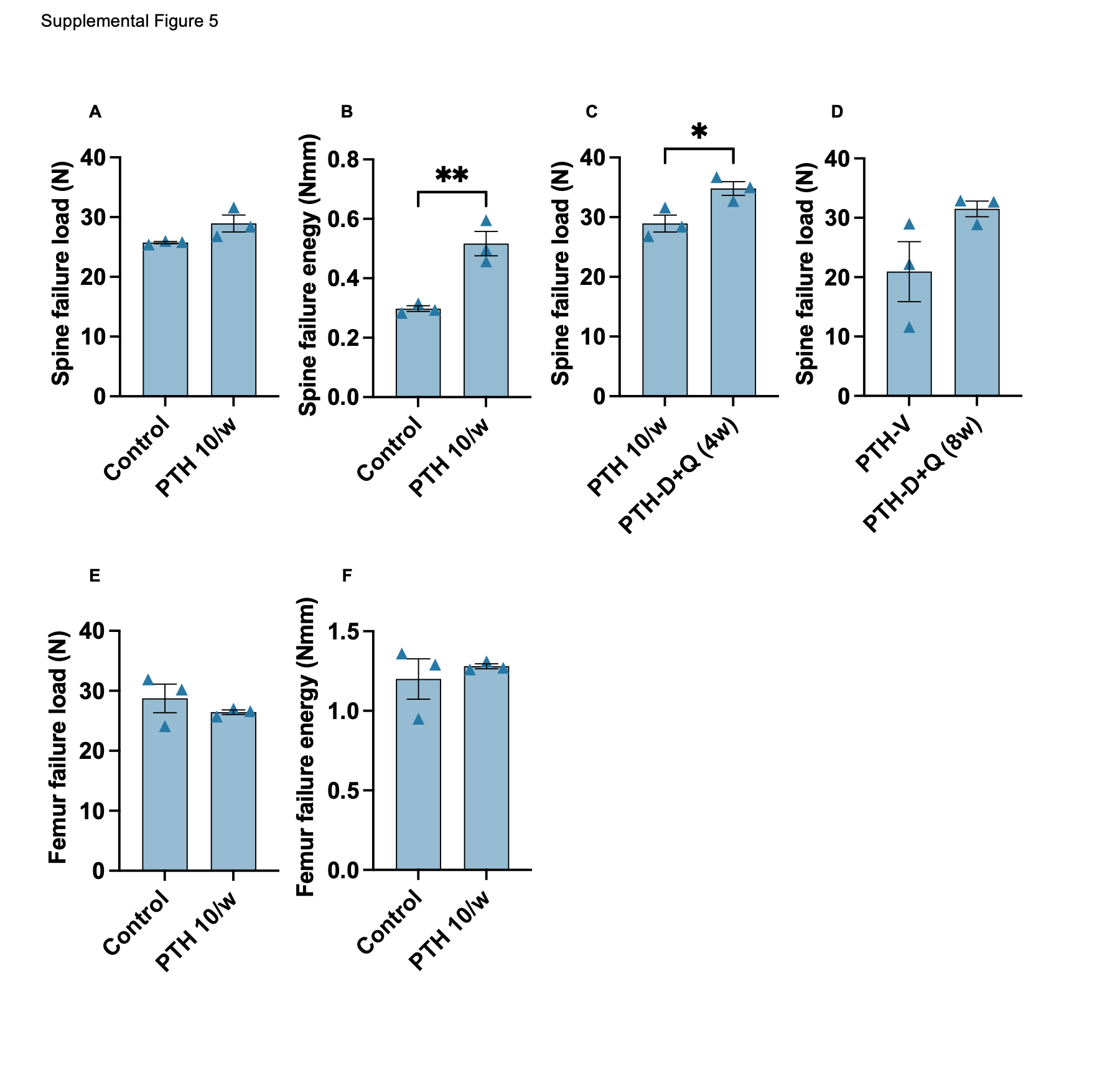

Supplement: Supplementary file 5 — Figure S5: Finite element analysis of aged mice. (A–D) Finite element analysis (FEA) result for failure load and failure energy of the spine (n = 3). Data represent mean ± SEM (error bars). *p < 0.05; **p < 0.01 (A, B: independent samples t‐test). SEM, standard error of the mean; PTH, D + Q, dasatinib + quercetin. [file ACEL-25-e70331-s002.tiff]

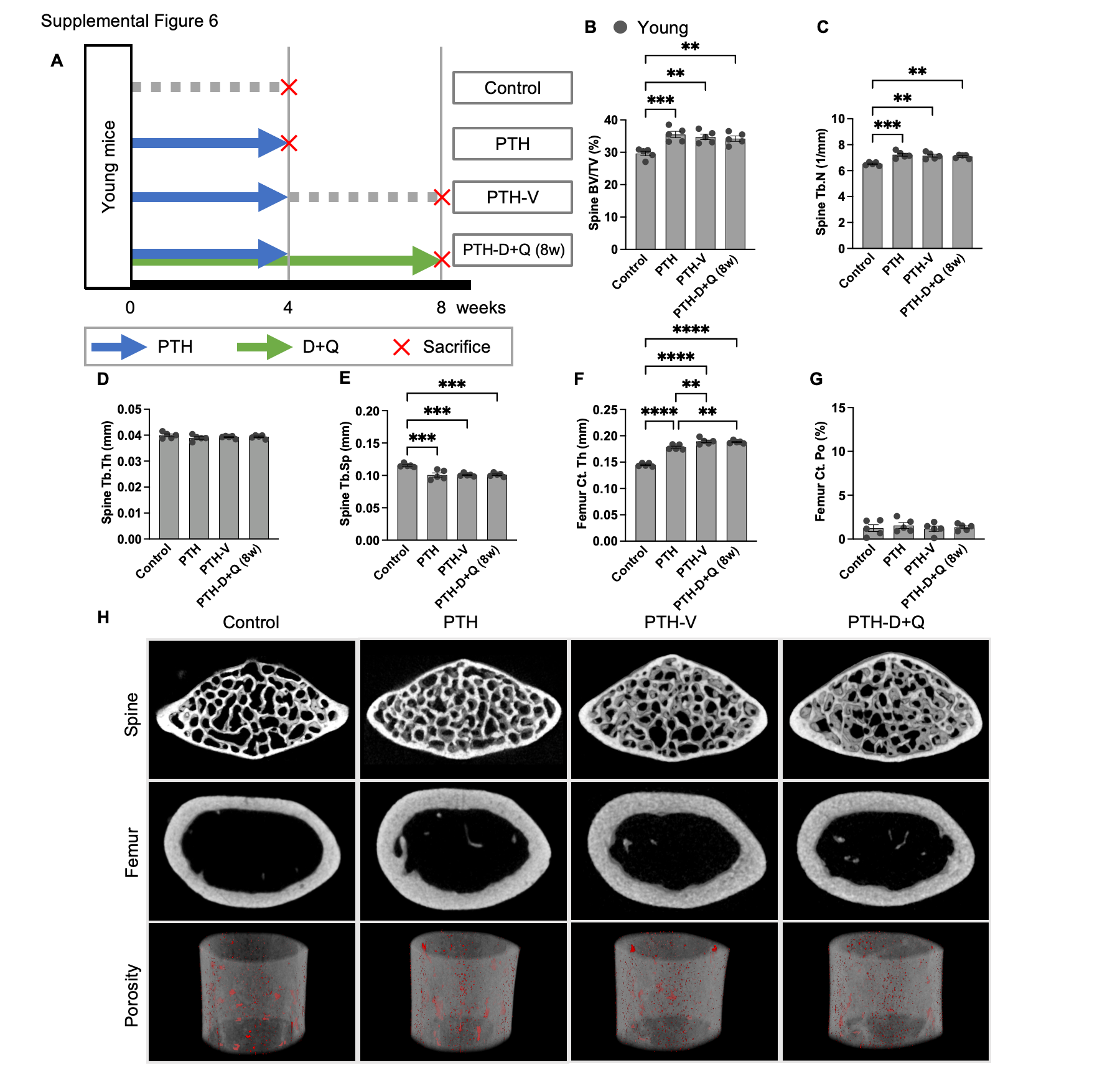

Supplement: Supplementary file 6 — Figure S6: Discontinuation of parathyroid hormone treatment or senolytic treatment caused no significant changes in the bone of young mice. (A) Experimental timeline illustrating the PTH treatment (10 times a week) and subsequent discontinuation, with or without D + Q co‐treatment. (B–E) Quantitative analysis of trabecular bone parameters (BV/TV, Tb. N, Tb. Th, and Tb. Sp) (n = 5). (F–G) Analysis of cortical bone parameters (Ct. Th, Ct. Po) (n = 5). (H) Representative micro‐CT images of the lumbar spine and femur in aged mice. Cortical porosity is highlighted in red (below). Data represent mean ± SEM (error bars). *p < 0.05; **p < 0.01; ***p < 0.001; ****p < 0.0001 (one‐way ANOVA with Tukey's multiple comparisons test). PTH, parathyroid hormone; D + Q, dasatinib + quercetin; BV/TV, bone volume; Tb. N, trabecular number; Tb. Th, trabecular thickness; Tb. Sp, trabecular spacing; Ct. Th, cortical thickness; Ct. Po, cortical porosity; CT, computed tomography; SEM, standard error of the mean; ANOVA, analysis of variance. [file ACEL-25-e70331-s008.tiff]

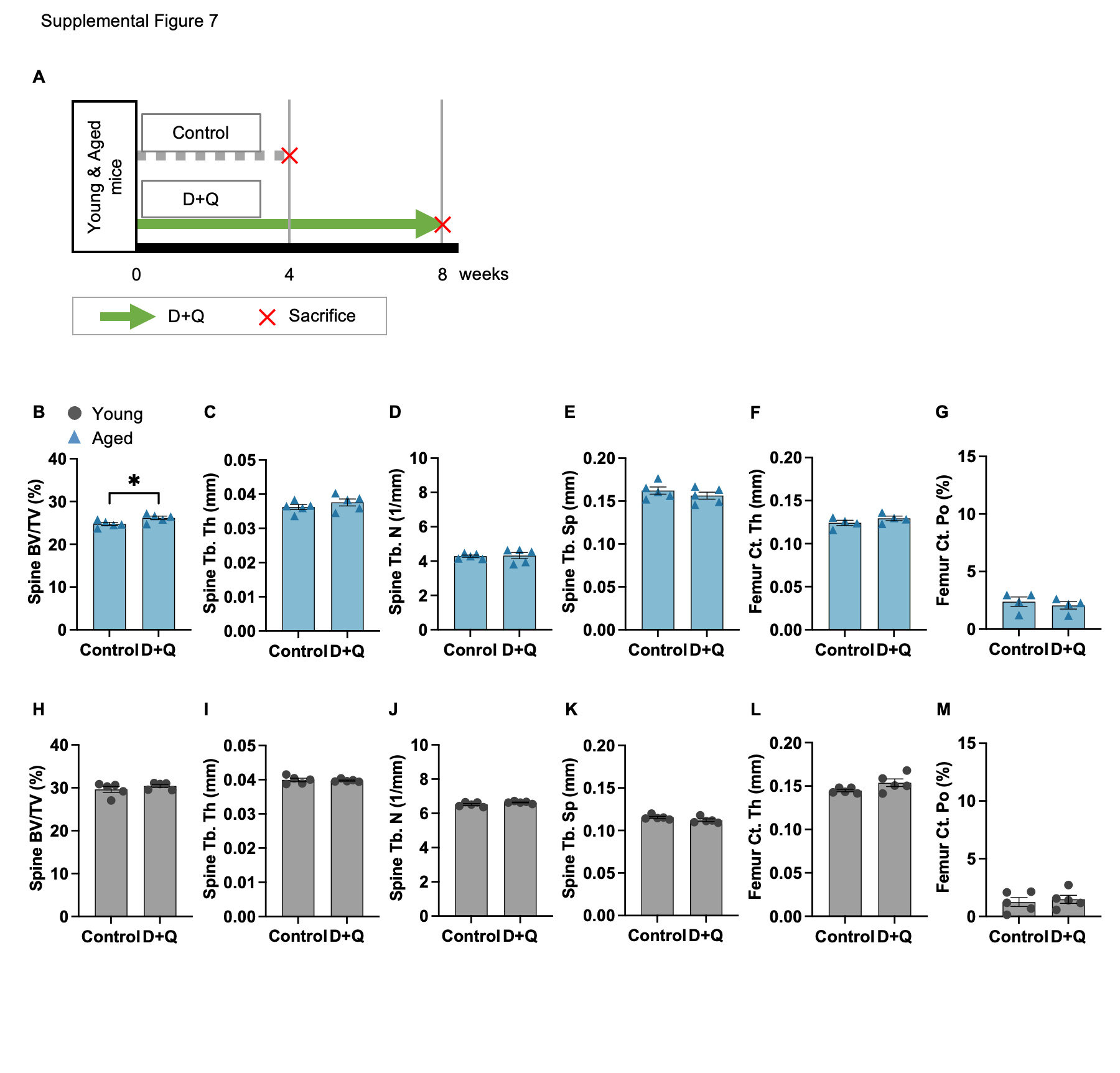

Supplement: Supplementary file 7 — Figure S7: Effects of 8 weeks of senolytic treatment (dasatinib + quercetin) on bone morphology. (A) Experimental design for D + Q treatment in young and aged mice. (B–E) Quantitative analysis of trabecular bone parameters (BV/TV, Tb. N, Tb. Sp, Tb. Th) in aged mice (n = 5 mice/treatment). Analysis of (F) cortical thickness (Ct. Th) and (G) cortical porosis (Ct. Po) in aged mice. (H–K) Quantitative analysis of trabecular bone parameters (BV/TV, Tb. N, Tb. Sp, Tb. Th) in young mice (n = 5 mice/treatment). Analysis of (L) cortical thickness (Ct. Th) and (M) cortical porosis (Ct. Po) in young mice. D + Q, dasatinib + quercetin; BV/TV, bone volume; Tb. N, trabecular number; Tb. Th, trabecular thickness; Tb. Sp, trabecular spacing. *p < 0.05; (independent samples t‐test). [file ACEL-25-e70331-s010.tiff]

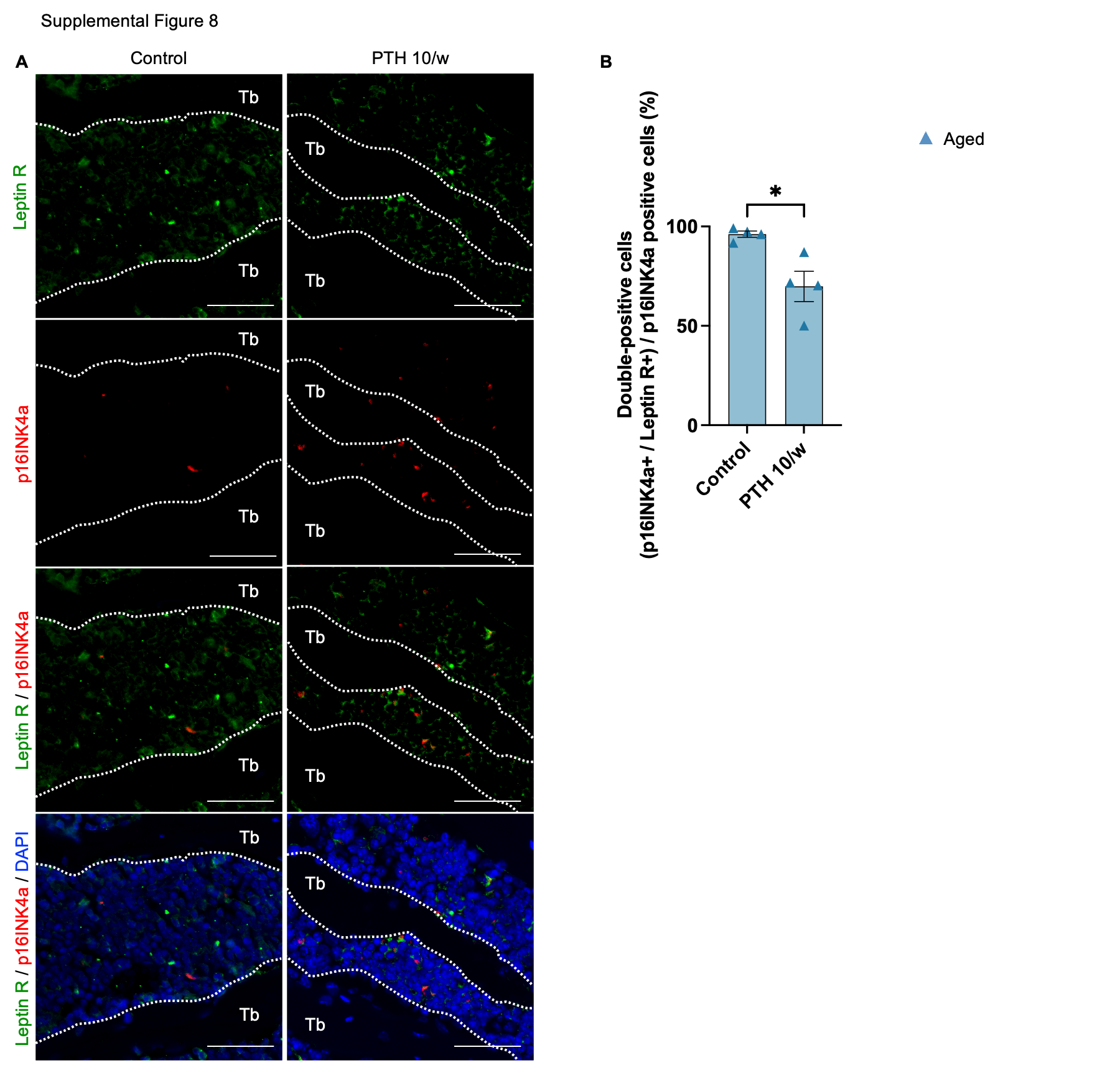

Supplement: Supplementary file 8 — Figure S8: Immunofluorescent co‐localization of leptin receptor and p16INK4a in bone tissue. (A) Double immunofluorescence staining for p16INK4a (red) and leptin receptor (Leptin R) in the lumbar spine of aged mice (Scale bars, 50 μm). (B) percentage of double‐positive cells (p16+/Leptin R+) per p16 positive cells (n = 4). *p < 0.05 (independent samples t‐test). PTH, parathyroid hormone. [file ACEL-25-e70331-s009.tiff]

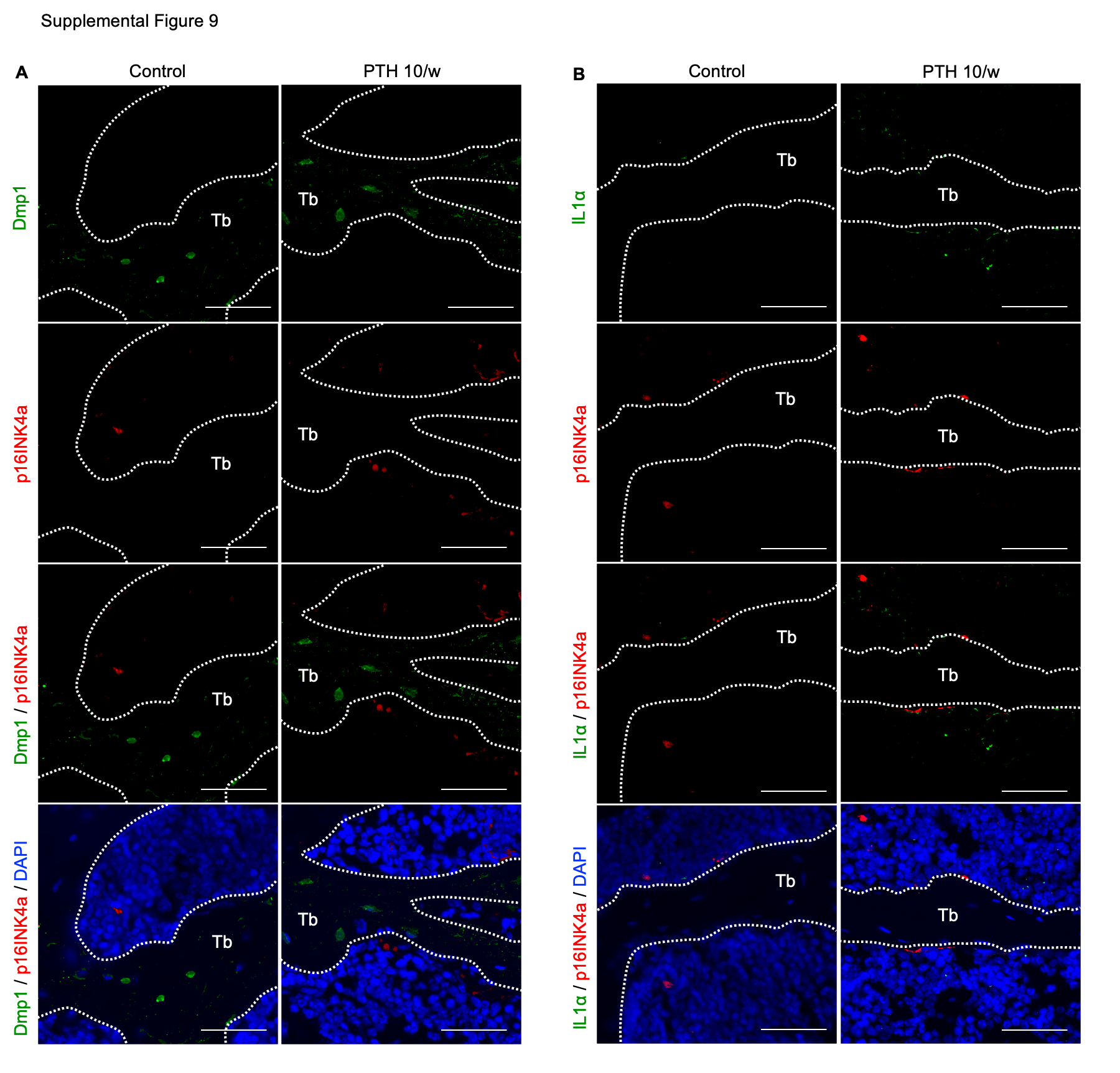

Supplement: Supplementary file 9 — Figure S9: Immunofluorescent co‐localization of Dmp1 and IL1α with p16INK4a. (A) Double immunofluorescence staining for p16INK4a (red) and Dentin matrix protein 1 (Dmp1) in the lumbar spine of aged mice (Scale bars, 50 μm). (B) Double immunofluorescence staining for p16INK4a (red) and Interleukin‐1 alpha (IL1α) in the lumbar spine of aged mice (Scale bars, 50 μm). DAPI, 4′,6‐diamidino‐2‐phenylindole; PTH, parathyroid hormone. [file ACEL-25-e70331-s001.tiff]
