# Supplementary material for: Senescent Cells Involved in Deterioration of Bone Microstructure by High‐Frequency Parathyroid Hormone 1–34 Administration and Bone Loss
Source: Aging Cell. 2025 Dec 23;25(1):e70331. doi: 10.1111/acel.70331 (PMC12723732; doi:10.1111/acel.70331)
Supplement: Supplementary file 10 — Table S1: Primers for RT‐qPCR. [file ACEL-25-e70331-s004.docx]

Supplemental Table 1. Primers for RT-qPCR

| Genes | Forward | Reverse |
| --- | --- | --- |
| *GAPDH* | 5’-CATCACTGCCACCCAGAAGACTG-3’ | 5’-ATGCCAGTGAGCTTCCCGTTCAG-3’ |
| *IL6* | 5’-TACCACTTCACAAGTCGGAGGC-3’ | 5’-CTGCAAGTGCATCATCGTTGTTC-3’ |
| *IL1α* | 5’-ACGGCTGAGTTTCAGTGAGACC-3’ | 5’-CACTCTGGTAGGTGTAAGGTGC-3’ |
| *IL1β* | 5’-TGGACCTTCCAGGATGAGGACA-3’ | 5’-GTTCATCTCGGAGCCTGTAGTG-3’ |
| *Mmp3* | 5’-CTCTGGAACCTGAGACATCACC-3’ | 5’-AGGAGTCCTGAGAGATTTGCGC-3’ |
| *Mmp13* | 5’-GATGACCTGTCTGAGGAAGACC-3’ | 5’-GCATTTCTCGGAGCCTGTCAAC-3’ |
| *Ccl5* | 5’-CCTGCTGCTTTGCCTACCTCTC-3’ | 5’-ACACACTTGGCGGTCCTTCGA-3’ |
| *Tnfsf11* | 5’-GTGAAGACACACTACCTGACTCC-3’ | 5’-GCCACATCCAACCATGAGCCTT-3’ |
| *Tnfrsf11b* | 5’-CGGAAACAGAGAAGCCACGCAA-3’ | 5’-CTGTCCACCAAAACACTCAGCC-3’ |
